# Nanosensor for the detection of eosin yellow and its photocatalytic degradation using phytosynthesized cerium oxide nanoparticles†

**DOI:** 10.1039/d4ra08231a

**Published:** 2025-02-06

**Authors:** Muhammad Usman Sadiq, Afzal Shah, Anum Zahid, Adeela Javaid, Faiza Jan Iftikhar

**Affiliations:** a Department of Chemistry, Quaid-i-Azam University Islamabad 45320 Pakistan afzals_qau@yahoo.com; b Department of Chemistry, Pir Mehr Ali Shah Arid Agriculture University Rawalpindi 46000 Pakistan; c Department of Chemistry, The University of Azad Jammu and Kashmir Muzaffarabad 13100 Pakistan; d National University of Technology (NUTECH) Islamabad 44000 Pakistan

## Abstract

A substantial rise in pollution due to population growth, industrialization, and urbanization has led to water contamination over the past few years, especially in developing countries. Dye-loaded effluents are often discharged into water reservoirs without any treatment, posing a significant threat to the sustainability of the environment and public health. Hence, it is imperative that researchers explore methods to effectively detect and eliminate dyes from waterways and suggest tangible solutions. With this consideration, the current work presents an efficient, cost-effective, eco-friendly, and sustainable approach for the detection of water pollutant (eosin yellow: EY) and its elimination from water. The voltammetric detection of EY was carried out on a transducer fabricated with carboxyl-functionalized multiwalled carbon nanotubes, while its removal was carried out using green-synthesized cerium oxide nanoparticles (CeO_2_ NPs). Under optimized conditions, the designed sensor detected EY up to a 1.24 nM limit of detection. CeO_2_ NPs were prepared using *Cassia fistula* leaves for the photocatalytic removal of EY from wastewater. The phytosynthesized CeO_2_ NPs were analyzed by X-ray diffraction analysis, photoluminescence spectroscopy, UV-visible spectroscopy, differential scanning calorimetry, scanning electron microscopy coupled with energy-dispersive X-ray spectroscopy, and Fourier transform infrared spectroscopy. These synthesized CeO_2_ NPs having a band gap of 3.24 eV were applied for the photocatalytic breakdown of EY under direct sunlight. Results revealed 99% removal of EY by CeO_2_ NPs from wastewater of pH 5. The findings of the current work emphasize the significance of nanosensor and nanomaterials-based photocatalysis for the detection of water contaminants and for devising a sustainable and environmentally benign remedial plan for water purification.

## Abstract 2

A substantial rise in pollution due to population growth, industrialization, and urbanization has led to water contamination over the past few years, especially in developing countries.

## Graphical abstract

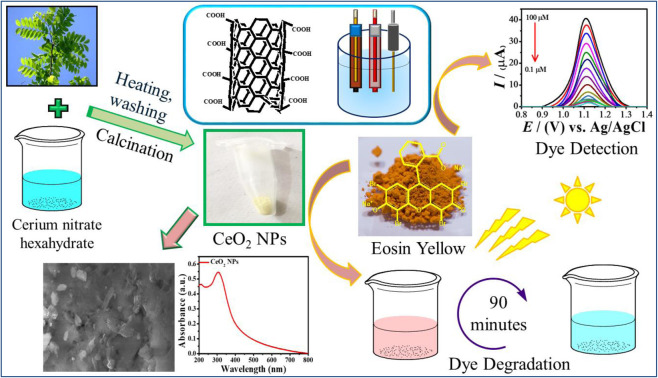

## 1. Introduction

The global population has soared to unprecedented levels, surpassing the 7.9 billion mark, and is still growing at a rate that has never been witnessed before. Estimates suggest that it will rise to 9.7 billion by the end of 2050.^1^ This rapid rise in population has led to immense pressure on the availability of resources and has increased the demand for basic human amenities such as health, food, and freshwater resources. The mismatch between the demand for resources and their dwindling supply has led to several challenges such as food and water scarcity, environmental degradation, and climate change. These issues need to be addressed through sustainable and responsible practices to respect the laws of the environment and ensure a secure future.^2,3^ At the heart of all these challenges, water contamination has emerged as a global issue with severe consequences for the environment and living beings. In this regard, the major global threat from industrial pollution is the dyes, which have been identified as the main xenobiotic contributors towards water contamination, disrupting photosynthesis and other biological activities of animals and plants.^4^ More specifically, dye contamination is resulting in a scarcity of clean water and food.

Chemical industries, including food, cosmetics, paper, and textile printing, use a variety of organic dyes for their product processing. Proper treatment and safe disposal regulations are often breached, and as a result, the majority of the dyes are released into water bodies untreated. Dyes are toxic to all forms of life as most of them are resistant to biodegradability and thus affect the food chain. Many organic dyes are capable of forming toxic chelation complexes with heavy metals in water.^5^ Furthermore, a variety of artificial dyes are popular due to their ability to add visually vibrant color to culinary dishes, which can be harmful when consumed beyond a certain limit.^6^ One such organic dye—Eosin Yellow (EY)—is a synthetic triarylmethane dye, commonly employed as a food and textile colorant. It can cause adverse effects such as pain, swelling, redness, and permanent damage to the eyes. EY along with its metabolites is carcinogenic and its consumption results in a harmful impact on essential organs such as kidneys, lungs, gastrointestinal organs, and liver.^7^ EY is banned for food usage in the United States due to its hazardous nature, yet it remains in high demand without any concerns about its risks in developing countries.^8^ Moreover, its untreated release into the environment poses toxic risks to both aquatic and human life. Hence, it is imperative that methods to effectively detect and eliminate these organic dyes from waterways be explored and developed. The current work is a contribution to this domain.

Most studies employ fluorescence methods, spectrophotometric analysis, and HPLC for the determination of EY. While these methods are known to be sensitive and accurate, they demand more time, require skilled personnel to handle, and involve complex preparation of the samples. Despite their effectiveness, more efficient, sensitive, easy-to-operate, and reliable methods are sought for the determination of EY to meet the requirements of environmental regulatory authorities. The adverse environmental effects associated with toxic dyes have prompted efforts toward the development of new and efficient dye detection and degradation methods. Electrochemical methods have shown great promise for the detection of dyes by offering the advantages of high sensitivity, selectivity, and rapid response time. Voltammetry and amperometry offer real-time monitoring for the prevention of contamination. In one particular study, Alghamdi and Kooli used hanging mercury drop electrodes for the sensitive detection of EY up to a 0.566 ppb concentration.^9^ In another report, Dhasarathan *et al.* employed carbon nanopowder-modified glassy carbon electrode (GCE) for the determination of EY up to 20 ppb using differential pulse voltammetry (DPV).^10^ In the same vein, our current work focuses on designing a sensitive platform for the detection of EY using carboxylic acid-functionalized multi-walled carbon nanotubes (COOH-fMWCNTs) immobilized onto a GCE.

COOH-fMWCNTs are extensively employed in electrochemical applications owing to their unique properties such as increased active sites, better dispersibility, improved electrocatalytic properties, and hydrophilicity.^11^ The functionalization with carboxyl groups introduces additional sites on the CNTs' surface, which can boost the electrochemical performance by promoting fast electron transfer and, at the same time, increase the molecular adsorption at the electrode surface.^12^ These unique characteristics find scope in applications such as supercapacitors, batteries, and sensors.^13^ Carboxyl groups improve the dispersibility of CNTs in different solvents including water. This is achieved by increasing the hydrophilicity of CNTs, which leads to better wettability with aqueous solvents and enhances ion transport at electrode–electrolyte interfaces.^14^ Thus, considering these merits, COOH-fMWCNTs were used in this work for nanomolar detection of EY.

Removal of the dyes from the environmental medium is vital and a hotly pursued area of research. Among different methods of water treatment (adsorption, chemical oxidation, coagulation, photocatalysis, and biological treatment), photocatalysis is an efficient, cost-effective, and versatile approach for environmental remediation.^15^ Different nanomaterials, including titanium dioxide, nickel oxide, zinc oxide, and tungsten oxide, have been employed for the photocatalytic removal of EY.^16–18^ However, currently, scientists are emphasizing the development of novel environment-friendly nanomaterials using green methods that can not only reduce the usage of toxic materials during their synthesis but also be efficient for wastewater treatment. CeO_2_ NPs have been shown to possess great potential to mitigate environmental pollution by degrading organic pollutants such as toxic dyes and drugs.^19,20^ CeO_2_ is an important rare earth n-type semiconducting material^21^ with a cubic fluorite lattice structure at the nanoscale range.^22^ These nano-CeO_2_ are known for their catalytic properties owing to a facile interconversion of Ce^3+^ and Ce^4+^, and the intrinsic existence of oxygen defects/holes in the crystal lattice that act as catalytic sites—the concentration of which increases as the material approaches the nano-regime.^23^

CeO_2_ NPs can be fabricated by spray pyrolysis, sol–gel, ball milling, hydrothermal, sonochemical, and solution precipitation methods.^24–27^ Despite the efficient control over size, morphology, and homogeneity, these approaches demand advanced equipment, more time for analysis, and high temperature and pressure conditions. They may also use toxic chemicals. While biosynthesis is a promising approach for the preparation of nanoparticles (NPs) without using any toxic chemicals, it is a suitable alternative to chemical methods.^28^ Therefore, the current work presents the green synthesis of CeO_2_ NPs by using leaf extract of *Cassia fistula* (*C. fistula*) for the photocatalytic removal of EY from water.

*C. fistula*, locally called Golden Shower or Amaltas, is a traditional medicinal plant that belongs to the family Leguminosae with medicinal applications as an anti-tumor, antifungal, hepato-protective, antimicrobial, and antioxidant agent.^29^ The leaf extracts are reported to be effective in the treatment of conditions such as piles, rheumatism, ulcers, jaundice, facial paralysis, and insect bites. These leaves are rich in flavonoids, anthraquinones, terpenoids, saponins, tannins, phenolic compounds, steroids, proteins, carbohydrates, and anthocyanosides.^30^ The presence of polyphenolic compounds such as *Rhein*, *Physcion*, *Kaempferol*, *Chrysophanol*, *etc.*, show that *C. fistula* leaves extract can be effectively used as a bio-reducing agent.^31^ The flavonoids undergo tautomerization from “enol” to “keto” and release hydrogen atoms, which result in the bio-reduction of metal ions to NPs. Moreover, phytochemicals possessing O–H groups (polyphenols) and N–H groups (amines) have a strong affinity towards metal ions and can act as reducing agents by donating electrons to metallic ions.^32^ Moreover, the presence of various anthraquinones and flavonoids allows the stabilization of the synthesized NPs and prevents their agglomeration.

In the present work, a COOH-fMWCNTs-based sensing platform was used for the detection of EY dye in an aqueous medium while *C. fistula* extract-mediated CeO_2_ NPs were used to effectively photodegrade the toxic dye. The green synthesized CeO_2_ NPs were investigated using Fourier transform infrared spectroscopy (FTIR), photoluminescence spectroscopy (PL), UV-visible spectroscopy (UV-vis), X-ray diffraction analysis (XRD), and scanning electron microscopy (SEM). Finally, the photocatalytic activity of biogenically fabricated CeO_2_ NPs was evaluated for efficient treatment of wastewater. The phytosynthesis of CeO_2_ NPs has been reported by using different plants, but still, there is no reported literature on the synthesis of CeO_2_ NPs using leaves extract of *C. fistula*. Based on the extensive survey of previous literature, this work reports for the first time an efficient method for the detection and degradation of EY using an environmentally friendly method. This work thus pioneers in presenting the effectiveness of an environmentally benign method for the elimination of the dye and also introduces an efficient method for its detection. The study will be useful for advancing a facile and straightforward detection method for the toxic dye EY that further contributes towards the removal strategy for contaminated wastewater.

## 2. Experimental

### 2.1 Materials

Cerium nitrate hexahydrate (Ce(NO_3_)_3_·6H_2_O), sodium hydroxide, hydrochloric acid, sulphuric acid, potassium nitrate, potassium chloride, sodium chloride, boric acid, phosphoric acid, disodium hydrogen phosphate dehydrate, potassium hexacyanoferrate, sodium dihydrogen phosphate monohydrate, ethanol, ethylenediamine tetraacetic acid (EDTA), COOH-fMWCNTs, ascorbic acid, methanol, and EY of analytical grades were utilized during this work. The solvent used for preparing all solutions was distilled water.

### 2.2 Instrumentation

Nicolet Summit FTIR spectrometer was used for recording the FTIR spectrum of CeO_2_ NPs to get information about surface functional groups, bonding, and molecular structure and to identify the functional groups of phytochemicals responsible for the stabilization and capping of these NPs during the synthesis route. Shimadzu 1700 was utilized to probe the optical properties of CeO_2_ NPs and their role as photocatalysts for the degradation of EY. PerkinElmer LS 55 spectrometer was employed for recording the luminescence spectrum. The differential scanning calorimetry (DSC) curves of the synthesized NPs were obtained using a Mettler Star SW 9.01 instrument at a heating rate of 10 °C min^−1^ in a −50 °C to 300 °C temperature range. The phytosynthesized CeO_2_ NPs were structurally analyzed by PANalytical X-ray diffractometer model 3040/60 X'Pert PRO operated at 45 kV and 40 mA. Copper's Kα radiation (*λ* = 0.154 nm) served as an X-ray radiation source applied at a step size of 0.025° over 2*θ* values ranging from 10°–80°. The surface morphology and elemental composition were analyzed by ZEISS field emission scanning electron microscope. Electrochemical investigations were made on Metrohm Autolab (galvanostat/potentiostat) from Utrecht, The Netherlands, which was operated with NOVA 1.11.0 software.

### 2.3 Transducer modification for the detection of EY

The GCE was initially cleaned on a nylon pad over water-alumina slurry to eliminate surface impurities. The surface was then washed with distilled water (DW) followed by sonication in a mixture of acetone, DW, and ethanol for 15 minutes to get rid of any contaminants acquired during polishing. After sonication, the electrode was dried at ambient conditions. For the modification of the clean surface of GCE, 1 mg/1 mL slurry of COOH-fMWCNTs was prepared using dimethylformamide (DMF). This required dissolution of COOH-fMWCNTs in DMF followed by ultrasonication till the formation of a homogenous suspension. The slurry was then drop-casted on the pre-cleaned GCE using a micropipette and dried under ambient conditions. The modified GCE was ready for immersion in the solution of EY for electrochemical investigations.

### 2.4 Preparation of extract from *C. fistula* leaves

The fresh leaves of *C. fistula* were collected from the trees in Islamabad, Pakistan. New fresh leaves were washed in tap water and then rinsed thoroughly with DW. Leaves were then air-dried in the dark at room temperature. Then the leaves are desiccated and crumbled into a powdery substance. Plant leaves extract was prepared by soaking 5 g plant leaves powder in 100 mL DW and maintained at 65–70 °C for about three hours with constant stirring, followed by filtration. The mixture was filtered after extraction and stored at 4 °C.

### 2.5 Phytosynthesis of CeO_2_ NPs for photodegradation of EY

**Fig. 1:**
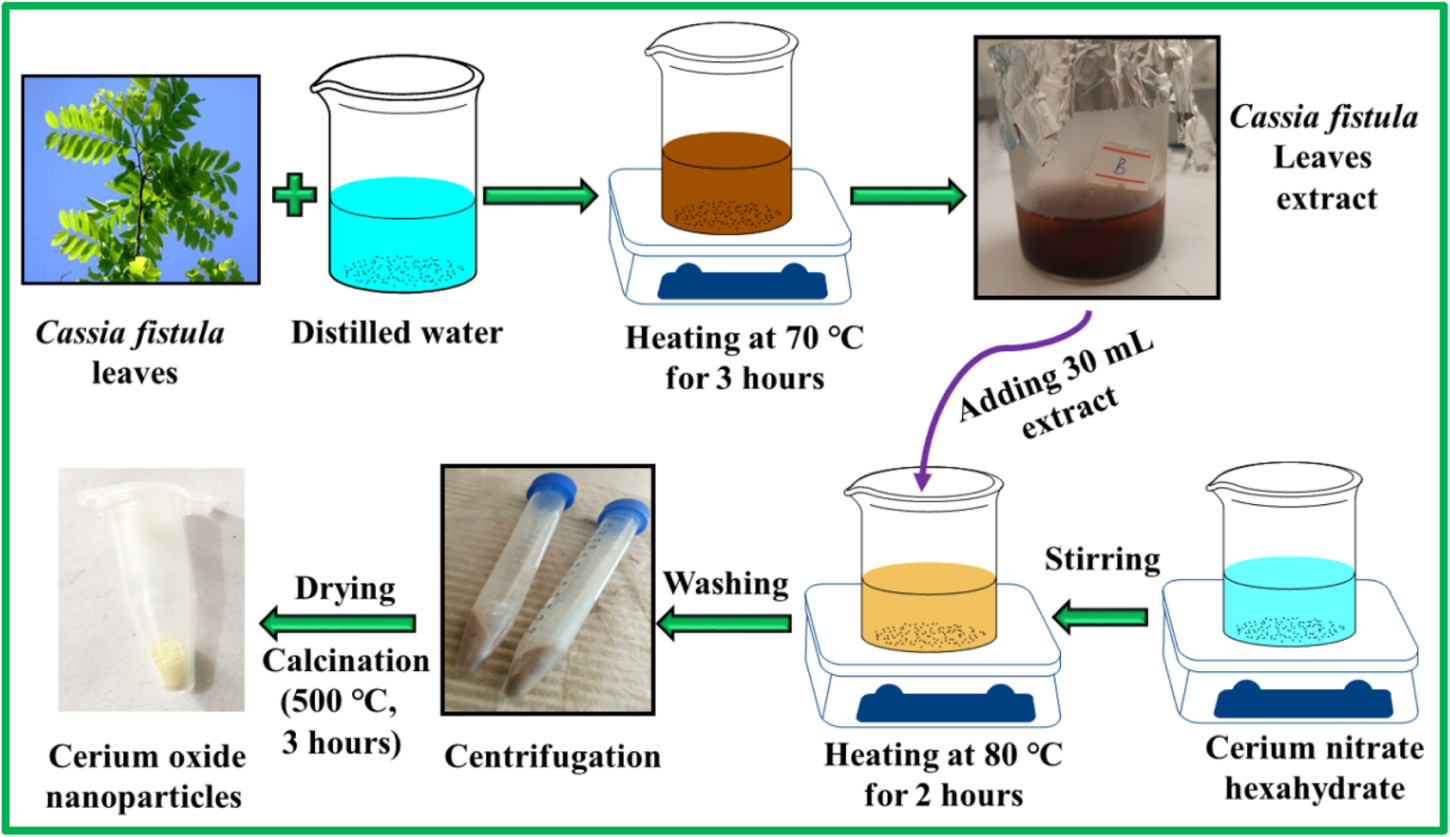
*C. fistula*-mediated synthesis of CeO_2_ NPs.

For phyto-fabrication of CeO_2_ NPs, 1 g of Ce(NO_3_)_3_·6H_2_O was added to 70 mL of DW and magnetically stirred to get a clear solution. Thereafter, 30 mL of *C. fistula* leaves extract was mixed with the salt solution. The temperature of the mixture was steadily raised to 80 °C and kept at the set temperature for 2 hours to obtain a light brown precipitate. This was followed by washing the precipitates with ethanol and DW (multiple times) by subjecting them to 5000 rpm centrifugation for 10 minutes. The obtained gel was then dried at 100 °C and fine powder was made by grinding. As a final step, the powdered sample was calcinated at 500 °C for 3 hours to achieve yellow-colored CeO_2_ NPs. The step-wise synthesis procedure of CeO_2_ NPs is illustrated in Fig. 1.

#### 2.5.1 Photocatalytic degradation procedure

The photocatalytic degradation of EY was spectrophotometrically examined by a UV-vis spectrophotometer. For the photocatalytic degradation experiment, 30 mL of 50 μM EY solution was exposed to direct sunlight irradiation for different periods of time. About 4 mL sample was taken from the dye solution exposed to sunlight at regular intervals and photocatalytic degradation was monitored by recording the UV-vis spectrum. Experiments were carried out with different parameters in order to determine the optimal solution pH and photocatalyst dosage necessary for maximum degradation efficiency. The degradation of EY was studied in a 4–8 pH range. For pH adjustment of EY solution, 0.1 M NaOH and 0.1 M HCl were used. Whereas, the impact of photocatalyst dose was studied by utilizing 2–10 mg of CeO_2_ NPs under optimized pH conditions. Before solar light illumination in each experiment, the dye solution was stirred under the dark for an half hour to achieve adsorption–desorption equilibrium. In addition, a radical scavenging experiment with ascorbic acid, methanol, and EDTA was carried out to investigate the real mechanism of the degradation process.

## 3. Results and discussion

### 3.1 Characterizations of CeO_2_ NPs

**Fig. 2:**
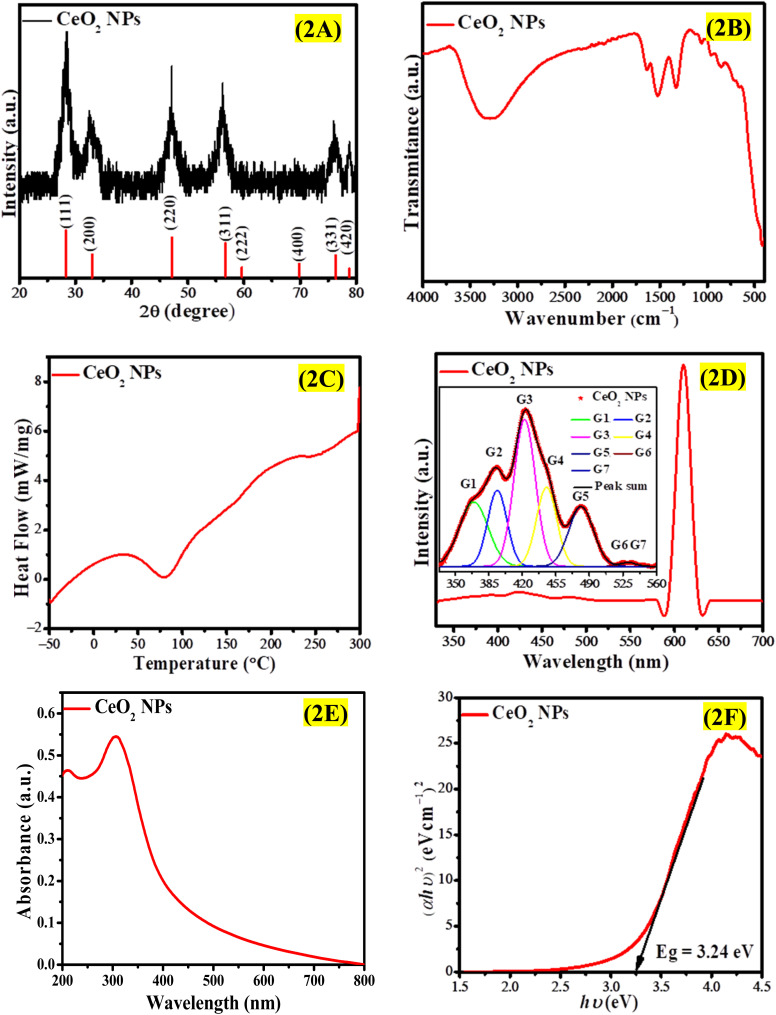
(A) XRD spectrum, (B) FTIR spectrum, (C) DSC curve, (D) PL emission spectrum (inset shows emission spectrum in the range of 330–560 nm), (E) UV-vis spectrum, and (F) Tauc plot for direct band gap of CeO_2_ NPs.

The nature of the phases and crystalline structure of the synthesized CeO_2_ NPs was determined through XRD analysis, and the spectrum (Fig. 2A) showed peaks at 28.33°, 32.80°, 47.12°, 56.16°, 59.62°, 69.80°, 76.43°, and 87.86°, which correspond to CeO_2_ with face-centered cubic structure.^25^ The results of the XRD pattern for synthesized CeO_2_ are in accordance with the JCPDS card no. 34-0394 corresponding to cubic fluorite structure.^33^ The intensity of peak is the highest for the (111) diffraction plane, while (222) and (400) planes have low peak intensity indicating that the (111) plane is more densely packed with atoms and is the dominant plane in the crystal lattice. The average crystallite size (*D*) assessed through the Debye–Scherrer formula, was found to be 5.44 nm, which is rather small in normal PV coordinates. This indicates a small crystallite size for NPs, whereas no additional peaks confirm the formation of a pure cubic phase of CeO_2_ NPs. Shanmugam *et al.*^34^ synthesized CeO_2_ NPs *via* the co-precipitation technique, employing sodium hydroxide and polyvinylpyrrolidone. The XRD analysis of the resulting CeO_2_ NPs indicated a high degree of crystallinity with a cubic phase, aligning well with JCPDS card no. 34-0394. Likewise, the XRD results for CeO_2_ NPs created through the pulse plasma in liquid method revealed a face-centered cubic fluorite structure, consistent with JCPDS card no. 34-0394.^35^

The FTIR spectrum of the phytosynthesized CeO_2_ NPs is depicted in Fig. 2B. A broad peak at 3298 cm^−1^ may be attributable to O–H stretching vibrations characterizing a hydroxyl group or residual water.^36^ The peak at 1326 cm^−1^ relates to NH stretching and the peak at 1538 cm^−1^ corresponds with CH stretching.^37^ These vibrational modes indicate that the phytochemicals are tightly capped with CeO_2_ NPs through amide and amines. The small peak at 1062 cm^−1^ originates from atmospheric CO_2_. The peak about 851 cm^−1^ and extensive band at 400–662 cm^−1^ are induced by O–Ce–O vibration.^38^ Fudala *et al.*^25^ synthesized CeO_2_ NPs through sol–gel method using ammonium hydroxide and urea. The FTIR analysis revealed the presence of six absorption bands at specific wavenumbers: 713 cm^−1^ corresponding to the Ce–O–C bond, 1111 cm^−1^ associated with C–N stretching vibrations, 1489 cm^−1^ indicative of ammonium ion deformation mode, 1543 cm^−1^ related to CO_2_ stretching vibrations, 1635 cm^−1^ linked to water bending vibrations, and 3412 cm^−1^ representing O–H stretching vibrations.

The synthesized CeO_2_ NPs were subjected to DSC analysis over the temperature range −50 °C to 300 °C to explore the thermal behavior and also confirm whether phytochemicals indeed had a role during NP synthesis. The DSC curve presented in Fig. 2C shows an endothermic peak in the range of 35–127 °C, which corresponds to completion of removal of phytochemicals adsorbed on NP surface. This phenomenon may also be attributed to the loss of water.^39^ An endothermic peak at 243 °C may be because of the decomposition of organic materials attached to NPs.^40^ The FTIR and DSC results confirmed a protective role of phytochemicals in the biogenic synthesis of CeO_2_ NPs. Baqer *et al.*^39^ prepared CeO_2_ using polyvinylpyrrolidone (PVP) as a capping agent through the heat treatment method. The DSC curve indicated an initial weight loss occurring at approximately 80 °C, attributed to the loss of water, while a subsequent weight loss around 440 °C was associated with the decomposition of PVP. Our results demonstrated a comparable pattern, with the first weight loss observed at 81 °C due to water loss or the removal of phytochemicals, followed by a second weight loss at 243 °C resulting from the decomposition of organic compounds.

PL spectroscopy is a promising technique to explore the optical characteristics of NPs. The room temperature emission spectrum of CeO_2_ NPs recorded at 305 nm (4.05 eV) excitation wavelength is depicted in Fig. 2D. The PL spectrum reveals an intense peak at 609 nm (2.03 eV) along with less intense peaks at 366 nm (3.38 eV), 392 nm (3.15 eV), 422 nm (2.93 eV), 445 nm (2.78 eV), 481 nm (2.57 eV), 522 nm (2.37 eV), and 537 nm (2.30 eV). The PL emission spectrum ranging from 330 nm to 560 nm is presented in the inset Fig. 2D to clearly illustrate the positions of emission peaks. The emission spectrum was resolved using Gaussian fitting and characterized as G1, G2, G3, G4, G5, G6, and G7. The UV emissions G1 (366 nm) and G2 (392 nm) are caused by the band recombination mechanism, which may involve free or localized excitons. The blue emissions G3 (422 nm) and G4 (445) can be ascribed to various defects, particularly dislocation, which facilitate rapid oxygen transfer. The blue-green emission G5 (481 nm) arises due to surface defects in CeO_2_ NPs. Two green emissions G6 (522 nm), and G7 (537 nm) can be linked to the oxygen vacancies in the crystal lattice of CeO_2_ NPs. Atmospheric O_2_ can react with these surface oxygen vacancies leading to highly reactive oxygen species over the NPs surface. This results in PL emissions at various wavelengths. The oxygen vacancies can trap the electrons, thus decreasing the chance of electron–hole recombination resulting in improved photocatalytic performance. The most intense emission peak at 609 nm may be related to the charge transfer transition from a higher 4f level (valence band) of Ce to a 2p level (valence band) of oxygen. It can also be ascribed to the incorporation of carbon from plant sources into the CeO_2_ NPs.^41,42^ The findings of this study align with existing literature. Minor discrepancies may arise from differences in synthesis methods, leading to the production of CeO_2_ NPs with varying sizes, shapes, and optical characteristics. Baqer *et al.*^39^ identified a violet light emission band at 410 nm, a broad blue emission band at 420 nm, and a blue-green band at 478 nm, which can be linked to the quantum size effect. Additionally, Sujana *et al.*^43^ reported that CeO_2_ NPs synthesized *via* the precipitation method and calcined at 800 °C and 400 °C exhibited a green band near 524 nm and a prominent violet light emission peak around 411 nm.

The photocatalytic role of NPs can be determined by analyzing their optical properties. The band gap value determined from UV-vis spectroscopy can be linked to the photocatalytic effect. In the case of material with a large band gap, fewer electrons will be excited to the CB and hence the activity of the material will be less. However, if the band gap is narrow, there may be an additional phenomenon *i.e.* the electron–hole recombination may also increase; thus, the activity will be decreased too. Therefore, an optimized value of band gap is desired for a specific application so that the generation of electron–hole pairs could be faster than their recombination. The optical absorption spectrum of CeO_2_ NPs showed maximum absorption signals at 210 nm and 305 nm, as evident from Fig. 2E, which corresponds to distinctive peaks of Ce^3+^ and Ce^4+^. The Tauc relation was used for the estimation of the band gap.^44^Fig. 2F shows that the direct band gap of CeO_2_ NPs is 3.24 eV. Fudala *et al.*^25^ identified an absorption signal at 300 nm while Wee *et al.*^45^ observed an absorption peak at 305 nm. These absorption peaks in this wavelength range occurred due to charge transfer between 2p of O^2−^ and 4f of Ce^4+^.

**Fig. 3:**
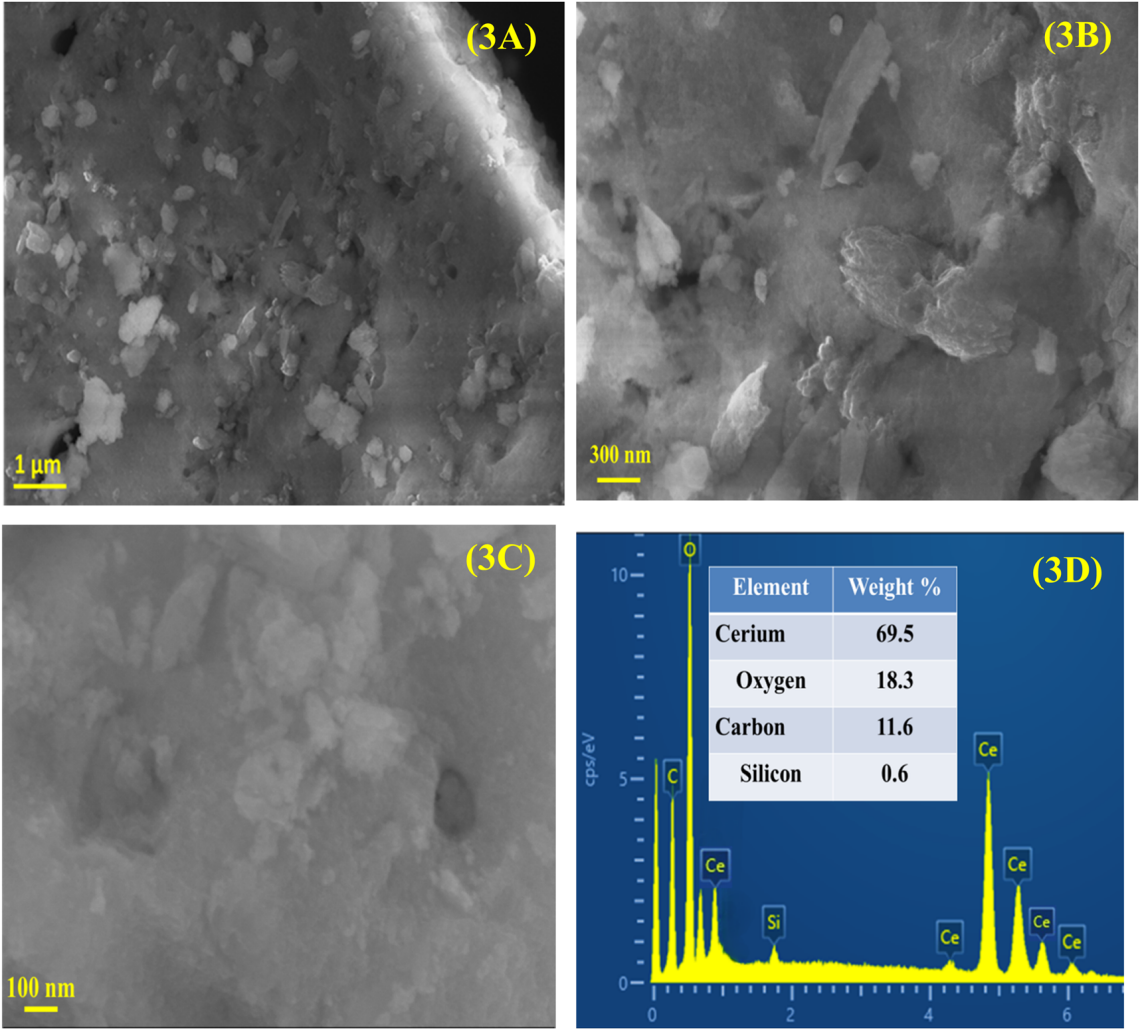
(A–C) FE-SEM micrographs of CeO_2_ captured at various magnifications along with (D) EDX analysis.

SEM micrographs of CeO_2_ NPs are displayed in Fig. 3A–C. These images indicate that CeO_2_ NPs have an irregular morphology and a non-uniform distribution. CeO_2_ NPs have been aggregated to have clusters like structures, which can be attributed to the reduction of surface energy as the preferable approach. Furthermore, the sample can be seen with many pores and voids, as a consequence of the combustion of organic moieties during the calcination process. These pores are the main reason why the surface area has been increased, thus, the photocatalytic performance has been improved. The sonochemical synthesis CeO_2_ NPs was carried out by Vatanparast and Saedi^46^ to investigate the effects of reaction conditions on the morphology and particle size using SEM. Varying concentrations of ethylenediamine (0.1–1.8 mL) demonstrated that up to 0.6 mL resulted in the formation of finer and more uniform particles, while concentrations of 1.8 mL caused aggregation and irregularity due to carbon combustion during annealing. The incorporation of hydrazine with ethylenediamine enabled controlled ion release, which enhanced nucleation and growth, whereas the absence of hydrazine resulted in the formation of larger particles. The introduction of NaOH during synthesis led to NPs aggregation due to the swift release of hydroxyl ions. Additionally, employing ultrasonic radiation during synthesis produced smaller particles, while its absence resulted in larger and non-uniform sizes. This study highlights the importance of optimizing reaction conditions for the synthesis of homogeneous and smaller NPs. Furthermore, the observed aggregation of NPs in this work indicates a higher concentration of phytochemicals in the plant extract utilized during the synthesis. The makeup of the phytosynthesized CeO_2_ NPs was analyzed *via* EDX which was then used to analyze the composition of the sample. EDX spectrum presented in Fig. 3D shows that CeO_2_ NPs are composed of Ce (69.5%), O (18.3%), C (11.6%), and Si (0.6%), which validates the purity of CeO_2_ NPs. The signal of C in the EDX spectrum may have been due to the inclusion of C during the synthesis process or it may be due to the C-tape/ribbon that was used to mount the sample during the analysis. The fact there is only 0.6% Si may be a result of contamination from the handling of the sample.

### 3.2 Electrochemical characterization of COOH-fMWCNTs/GCE

**Fig. 4:**
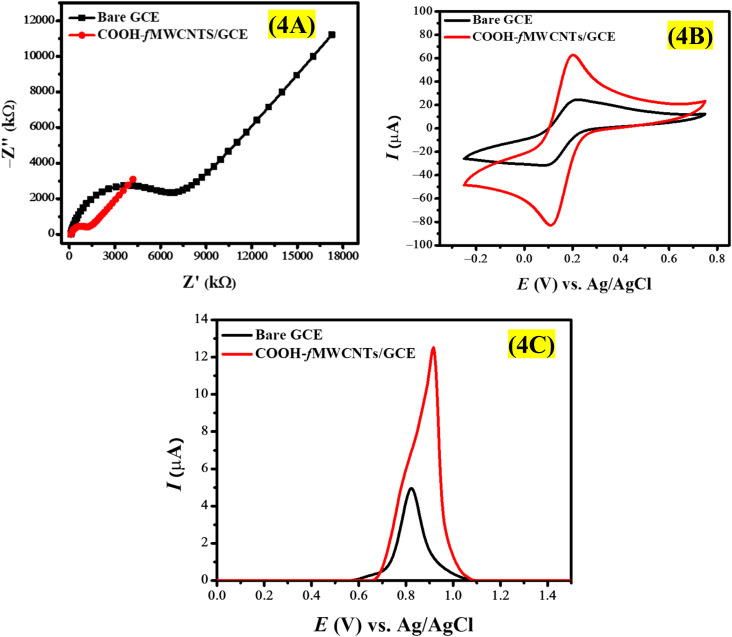
Comparison of the (A) Nyquist curves (B) cyclic voltammograms and (C) SWV at the bare and COOH-fMWCNTs/GCE.

EIS was employed to shed light on the electrical properties of the electrode–electrolyte interface clarifying the influence of the electrical resistance at the GCE surface. This work investigates the conductance differences between a COOH-fMWCNTs modified electrode and a bare electrode in a potassium ferricyanide solution through impedance spectroscopy. EIS spectra were obtained across a frequency range of 0.1 Hz to 0.1 MHz, utilizing an AC voltage with an amplitude of 10 mV (rms) and a DC voltage set at 0 V. The impedance spectra obtained with both the COOH-fMWCNTs modified electrode and the bare electrode in the potassium ferrocyanide solution were different from each other. The modified electrode exhibited a lower impedance magnitude, indicating higher conductivity in relation to the bare GCE (Fig. 4A).

The bare GCE displays a prominent semicircular region, signifying a high charge transfer resistance (*R*_ct_) due to insufficient active sites for electron transfer. The diameter of this semicircle in EIS is indicative of *R*_ct_, which measures the efficiency of electron transfer at the electrode-solution interface. A high *R*_ct_ value typically reflects diminished electrochemical reactivity, a characteristic of unmodified GCEs. Conversely, the COOH-fMWCNTs/GCE exhibits a considerably smaller semicircle, suggesting a marked reduction in *R*_ct_. This lower impedance points to improved electron transfer kinetics at the electrode surface, which can be attributed to the enhanced conductivity and increased surface area of the COOH-fMWCNTs/GCE. The functionalization process introduces additional reactive sites, facilitating quicker charge transfer and consequently lowering *R*_ct_. Furthermore, the COOH-fMWCNTs/GCE demonstrates a slightly elevated double-layer capacitance (*C*_dl_) compared to the bare GCE, likely due to the larger effective surface area and improved surface roughness resulting from the COOH-fMWCNTs. This expanded surface area allows for greater charge accumulation, thereby enhancing *C*_dl_. Additionally, Warburg impedance (*Z*_w_), which relates to diffusion-controlled processes where ions move through the electrolyte to the electrode interface, is higher for COOH-fMWCNTs/GCE (171.9 μSs^1/2^) than for the bare GCE (135.9 μSs^1/2^). The observed increase in *Z*_w_ can be linked to the highly porous nature of COOH-fMWCNTs, which may facilitate ion capture during their transit, thereby enhancing *Z*_w_. Additionally, the elevated *C*_dl_ of COOH-fMWCNTs/GCE could contribute to a more concentrated ionic layer at the surface, which may slightly impede ion diffusion due to heightened interactions within this layer.

The alpha (*α*) is an important parameter of the constant phase element in EIS that quantifies the extent of non-ideal capacitance at the electrode interface or surface heterogeneity. Its values range from 0 to 1, with a value approaching 1 indicating a more uniform surface, while a lower value signifies greater surface heterogeneity. The measured *α* values of 0.79 for bare GCE and 0.88 for COOH-fMWCNTs/GCE indicate that the modified GCE exhibits a more homogeneous surface compared to the bare GCE. These findings demonstrate that the addition of COOH-fMWCNTs increases the electrical conductivity of the electrode interface. The modified electrode has lower impedance which implies that the charge transfer kinetics are improved and the conductivity is enhanced. Thus, the positive role of COOH-fMWCNTs in the electrochemical performance is confirmed. The impedance data was analyzed and an equivalent circuit model was used to obtain deeper insights into the electrical processes. The impedance response of the modified electrode was fittingly characterized by a Randles' circuit shown in Fig. S1,† illustrating the effect of COOH-fMWCNTs on the charge transfer kinetics. The retrieved impedance data is given in Table S1† which reveals the fact that the reduced charge transfer resistance is related to the functionalization of GCE with COOH-fMWCNTs. On the GCE surface, COOH-fMWCNT film improves the electron transfer process between the analyte (K_3_[Fe(CN)_6_]) and the transducer.

The electrode surface area is a crucial factor, especially the electroactive surface area (SA) which was determined using cyclic voltammetry. Fig. 4B shows the voltammograms which are presented by using redox probe 5 mM K_3_[Fe(CN)_6_] in 0.1 M KCl solution. The Randles–Sevcik equation given in eqn (1) used to derive the value of the electroactive surface of the electrode.

1 *I*_pa_ = 2.6 × 10^5^*n*^3/2^*D*^1/2^*Cν*^1/2^*A*

In this equation, *n* represents the number of electrons (*n* = 1), *I*_pa_ the anodic peak current, *D* the diffusion coefficient (*D* = 7.6 × 10^−6^ cm^2^ s^−1^), *C* the molar concentration (*C* = 5 mM), *ν* the scan rate (*ν* = 100 mV s^−1^), and *A* the electroactive SA. As per calculations, the electroactive surface area for modified electrode was found to be 0.06 cm^2^, which is threefold greater that the SA of the bare electrode.

### 3.3 Voltammetric analysis of EY

Voltammetric analysis of EY was conducted with Britton Robinson buffer (BRB) solution (0.1 M, pH 7) in the potential range of 0 V to 1.5 V. Fig. 4C represents the voltammetric response of 100 μM EY on bare and COOH-fMWCNTs/GCE. According to Fig. 4C, COOH-fMWCNTs/GCE have a greater effect on the oxidation of EY, which leads to a distinct increase in the peak current. The signal was strengthened because of the higher electroactive area and better conductivity of COOH-fMWCNTs/GCE in relation to the bare GCE. A more active electroactive area of COOH-fMWCNTs/GCE increases the adsorption of EY while its superior conductivity allows the distance between the electrode and the analyte to be small enough which is an efficient electron transfer pathway for EY oxidation. Also, the conjugated structure of EY dye and COOH-fMWCNTs can interact through π–π interactions which also help the oxidation of EY. Consequently, an increased current response is observed.

#### 3.3.1 Optimization of voltammetric parameters

Choosing appropriate conditions for detection is essential to enhance the sensor's sensitivity to the targeted analyte. In order to boost the sensitivity of COOH-fMWCNTs/GCE for EY, various parameters were examined to identify the optimal conditions for its voltammetric analysis.

Selecting an appropriate inert electrolyte is crucial for the analysis of the desired analyte. Changes in the electrolyte can affect ionic strength, ohmic drop, and ionic migration, all of which can significantly influence the voltammetric results. The electro-oxidation of EY was investigated using COOH-fMWCNTs/GCE across various supporting electrolytes, such as phosphate buffer (PBS pH = 7), 0.1 M HCl, 0.1 M H_2_SO_4_, BRB pH = 7, citrate buffer saline (CBS pH = 5), 0.1 M NaOH, 0.1 M KCl, and 0.1 M NaCl. Fig. S2A† illustrates how different electrolytes affect the shape and intensity of the EY signal. It is evident from Fig. S2B† that the choice of electrolyte notably impacts both the shape and current of EY, with the highest current response observed in 0.1 M H_2_SO_4_. This acid is recognized for its excellent ionic conductivity, minimal interference with electrode materials, and stability over a broad potential range. Consequently, H_2_SO_4_ has been chosen for the subsequent electrochemical investigation of EY.

The concentration of supporting electrolytes plays a significant role in influencing the overall sensitivity of the chosen sensor. To investigate how H_2_SO_4_ concentration affects the current response of EY, voltammograms were obtained using varying concentrations of H_2_SO_4_ (Fig. S3A†). The results showed that the current response increased until reaching a concentration of 0.2 M. Beyond this point, there was a gradual decrease in the peak current of EY (Fig. S3B†). This decline is attributed to ion overcrowding, which causes an increase in background current, thereby diminishing the current signal from EY. The reported literature indicates that subjecting GCE to electrochemical treatment within a potential range of 0–1.8 V over multiple scans in the presence of H_2_SO_4_ promotes the oxidation of carbon atoms on the GCE surface. This process results in the formation of several oxygen-containing functional groups, including carboxyl, carbonyl, hydroxyl, and quinoid. The presence of these functional groups enhances the electrochemical performance of GCE, especially in the context of analyte detection.^47^ To determine whether the observed increase in the EY response is attributed to the functionalization of the GCE or the catalytic effect of COOH-fMWCNTs/GCE, three voltammograms of EY were obtained using a bare GCE in a 0.2 M H_2_SO_4_ supporting electrolyte. The data presented in Fig. S4† indicates that the current response of EY with the bare GCE is significantly lower than that with COOH-fMWCNTs/GCE, confirming that the enhancement in current is due to the catalytic properties of COOH-fMWCNTs. Additionally, Fig. S4† illustrates a significant shift in the oxidation potential of EY; it occurs at 1.22 V on the bare GCE, while it is reduced to 1.1 V with COOH-fMWCNTs/GCE. This demonstrates that the incorporation of COOH-fMWCNTs/GCE not only enhances the current response of EY but also facilitates the oxidation process.

Deposition influences how effectively the target analyte is adsorbed onto the modified electrode, which is crucial for evaluating the sensitivity of the sensor. By fine-tuning the deposition potential, the modified surface of the glassy carbon electrode can effectively draw in the target analyte, enhancing the sensor's performance. Analyzing the effects of varying accumulation potentials between 0 V and 0.6 V revealed that applying a more positive potential resulted in a marked increase in current, peaking at 0.3 V, as illustrated in Fig. S5A.† However, current levels began to diminish beyond 0.3 V. Consequently, a deposition potential of 0.3 V was determined to be optimal. The elevated peak current response at this voltage can be linked to the anionic nature of EY, which fosters a stronger and more effective interaction with COOH-functionalized multi-walled carbon nanotubes on the glassy carbon electrode. Furthermore, the duration of deposition plays a vital role in influencing the sensitivity of the modified GCE. The effect of accumulation time on the current response of EY was investigated by adjusting the deposition time from 5 seconds to 15 seconds, as illustrated in Fig. S6A.† The highest current signal was recorded at a deposition time of 5 seconds, as shown in Fig. S6B.† Extending the deposition time leads to a decrease in the peak current of EY. This can be attributed to the greater solubility of the dye in the supporting electrolyte. As a result, a longer deposition time may limit the quantity of dye available for oxidation at the modified electrode. Consequently, the ideal conditions for the voltammetric analysis of EY were determined to be a 0.2 M H_2_SO_4_ solution with a deposition potential of 0.3 V and a deposition duration of 5 seconds.

**Fig. 5:**
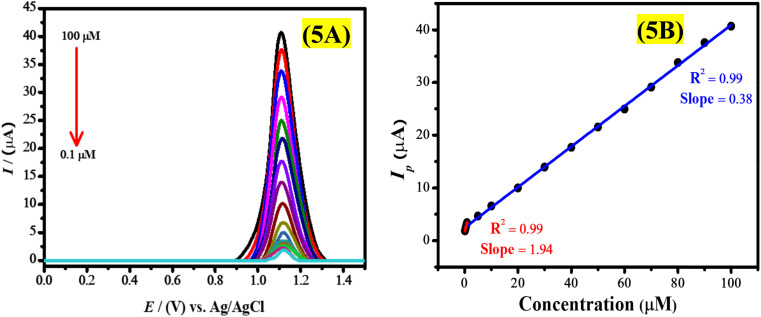
(A) Voltammetric response of various concentrations of EY using COOH-fMWCNTs/GCE and (B) linear plot using peak current values of 0.1 μM to 0.9 μM solutions of EY.

In numerous sectors, including environmental monitoring, pharmaceuticals, and food safety, there are regulatory mandates to identify and measure pollutants, contaminants, and pharmaceuticals at minimal concentrations as well as at defined levels. Establishing the Limit of Detection (LOD) is crucial to ensure that the analytical method complies with these regulations. The sensitivity of the modified electrode was assessed by performing Square Wave Voltammetry (SWV) on a range of ethyl yellow concentrations, from 0.1 μM to 100 μM, under optimized conditions: 0.2 M H_2_SO_4_, a 0.3 V accumulation potential, and a 5 seconds accumulation time. Fig. 5A demonstrates a decline in current response accompanied by a narrowing of the peaks as the concentration decreases. A linear calibration plot was created using the lower concentration values to determine the LOD, presented in Fig. 5B. The LOD and LOQ were calculated using the specified equations.^10^2
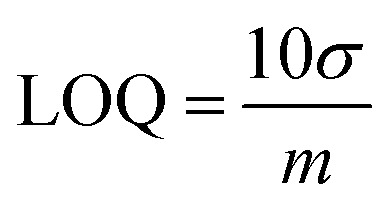
3
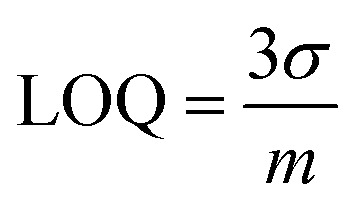


The standard deviation (*σ*) that was calculated from the anodic peak current of the blank solution (eleven runs) and the slope (*m*) was determined from the calibration plot conducted for whole concentration range and lower concentrations. The LOD with a value of 1.29 nM was obtained for the lower concentration range, whereas it was measured at 6.6 nM for the complete concentration range. Additionally, the LOQ was identified as 4.25 nM for lower concentrations and 22.03 nM for the full concentration range, respectively.

The stability of the COOH-fMWCNTs/GCE-based sensor was evaluated over a period of three days under optimized conditions. The current response remained consistent, showing no significant deviation from that of the freshly modified electrode (Fig. S7†).

#### 3.3.2 Scan rate effect on the voltammetric response of EY

The electrochemical behavior of a material plays a vital role in assessing its electrocatalytic properties. The increased peak intensity observed with a rising scan rate (*ν*) indicates that the electron transfer process is affected by factors like adsorption, diffusion, or a combination of both. To investigate the EY oxidation process, cyclic voltammograms were obtained by altering the *ν* from 20 mV s^−1^ to 120 mV s^−1^ using a 0.2 M H_2_SO_4_ solution. As shown in Fig. S8A,† the peak current (*I*_p_) increases linearly with the scan rate, without any noticeable reduction peak, which confirms the irreversibility of the EY oxidation reaction. To further analyze the characteristics of the EY oxidation reaction, linear plots were created relating *I*_p_ to *ν*, the logarithm of *I*_p_ to *ν*, and *I*_p_ to *ν*^1/2^, as illustrated in Fig. S8B–D.† The slope of the log *I*_p_*versus* log *ν* graph is 0.96, indicating that the EY oxidation process occurring on the surface of COOH-fMWCNTs/GCE is governed by adsorption. Additionally, the plot of *I*_p_*versus ν* shows a high correlation coefficient of 0.99, which, when compared to the *R*^2^ values derived from the *I*_p_*versus ν*^1/2^ graph, further supports the conclusion that the oxidation of EY is indeed controlled by adsorption.

### 3.4 Photocatalytic degradation of EY

The experiments for the photocatalytic degradation of EY involved adding 2 mg of CeO_2_ nanoparticles to 30 mL of EY solution in a neutral pH setting. This mixture was subsequently kept in the dark and stirred for thirty minutes to establish adsorption–desorption equilibrium. Following this, the EY solution was exposed to solar light. A small aliquot (approximately 4 mL) of EY was extracted from the dye solution at 10 minutes intervals, and the photodegradation was assessed by capturing the UV-vis spectrum. As shown in Fig. S9A,† a reduction in EY absorbance was observed, indicating the rate of photodegradation. The efficiency of photocatalytic degradation was calculated using the subsequent equation.4
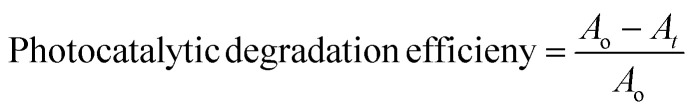
In a neutral pH environment, the photocatalytic degradation of EY reached 66% with the application of 2 mg of CeO_2_ nanoparticles. The rate constant (*k*) was calculated to be 0.0117 min^−1^, utilizing a first-order kinetic equation and plotting ln(*A*_*t*_/*A*_o_) against time (*t*).5
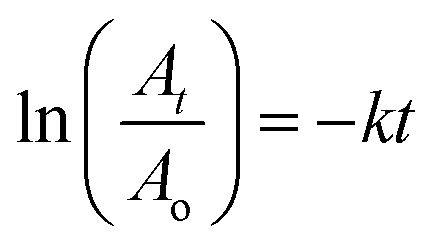


#### 3.4.1 Optimization of the photocatalytic degradation conditions

This study examined the effects of pH and the dosage of CeO_2_ nanoparticles on the photodegradation of EY, aiming to identify the optimal conditions for the most effective removal of dye from water.

##### 3.4.1.1 Impact of pH on the photocatalytic degradation of EY

A significant aspect to consider is the pH of the solution, which plays a vital role in regulating the photocatalytic efficiency of a catalyst. Changes in pH can lead to the protonation or deprotonation of the redox properties of the dye structure, thereby affecting the dyes' vulnerability to photodegradation. The effect of pH fluctuations is largely contingent upon the chemical composition of the dyes, particularly for those containing charged molecules, where the impact is most pronounced. pH variation significantly affects the surface characteristics of a catalyst, including particle aggregation, band edge position, and surface charge. Typically, acidic conditions can lead to the protonation of the photocatalyst surface, resulting in a positively charged catalyst, while basic conditions produce a negatively charged catalyst. Consequently, in acidic environments, anionic dyes are strongly attracted to positively charged photocatalysts, facilitating rapid degradation. In contrast, the degradation of cationic dyes is less effective due to electrostatic repulsion, resulting in a slower degradation rate. However, in basic conditions, the photodegradation of cationic dyes becomes more efficient due to the strong interaction between the dye molecules and negatively charged photocatalysts.

The effect of pH on the photocatalytic degradation of EY was investigated through experiments conducted within a pH range of 4 to 8, utilizing 30 mL of a 50 μM EY solution and a 2 mg dosage of CeO_2_ nanoparticles. This study monitored the photodegradation of EY solutions at various pH levels over a period of 2 hours to assess the influence of pH on degradation efficiency. The UV-vis spectra presented in Fig. S10† indicate that the photodegradation of the EY solution was enhanced in acidic conditions, with optimal degradation occurring at pH 5, as illustrated in Fig. S10B.† Conversely, a reduction in photodegradation efficiency was noted in a basic environment (pH 8), as shown in Fig. S10E.† In acidic conditions, the protonation of the CeO_2_ surface leads to a positively charged state, which effectively attracts anionic EY, facilitating efficient photodegradation. Conversely, in alkaline environments, the deprotonation of CeO_2_ results in a negatively charged surface that repels EY molecules, thereby hindering photodegradation. It was noted that the photodegradation of EY improved from 66% at pH 7 to 93% at pH 5, with optimal degradation occurring at pH 5.

**Fig. 6:**
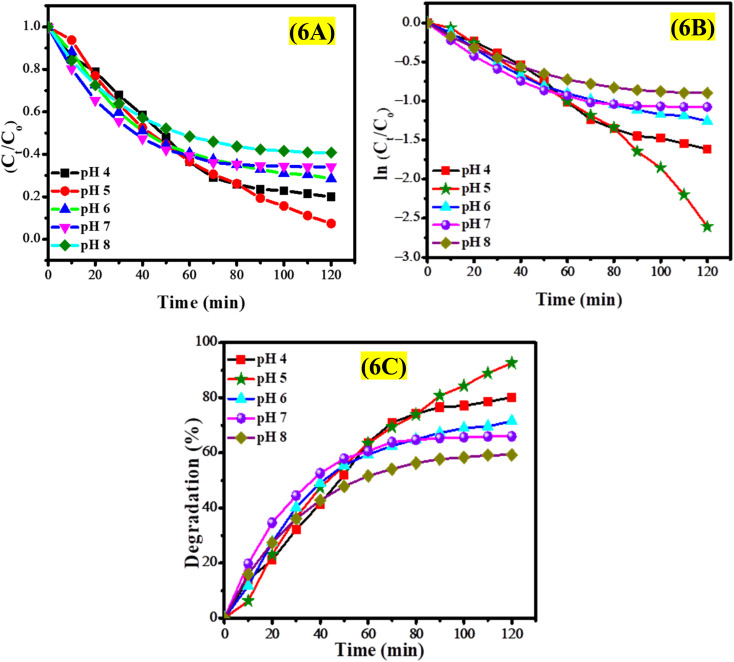
(A) Graph of *C*_*t*_/*C*_o_*versus* time, (B) kinetic analysis of the photocatalytic degradation of EY in different pH environments, and (C) impact of solution pH on the photocatalytic EY degradation efficiency of CeO_2_ NPs.

The degradation profile of EY across various pH levels is depicted in Fig. 6A. The findings reveal a strong dependence of degradation behavior on pH, with the fastest photocatalytic degradation occurring at pH 5, indicating that mildly acidic conditions are optimal for degradation. Conversely, the slowest degradation was noted at pH 8, highlighting the increased stability of EY in basic environments and the diminished electrostatic interaction between the negatively charged CeO_2_ nanoparticles and the anionic EY dye. A pseudo-first-order kinetic model was employed to analyze the kinetics of EY degradation, as depicted in Fig. 6B. The rate constants calculated for the photocatalytic degradation of EY at various pH levels are provided in Table S2.† The degradation percentage of EY solutions under different pH levels is illustrated in Fig. 6C, indicating rapid degradation in the medium of pH 5.

CeO_2_ nanoparticles exhibit modifications in their surface properties that are influenced by the pH of the environment. In acidic conditions (pH 4–6), the surface of CeO_2_ nanoparticles becomes protonated, resulting in a positively charged surface that effectively attracts negatively charged EY through electrostatic interactions. This attraction enhances the adsorption of EY onto the CeO_2_ nanoparticles, thereby promoting efficient photocatalytic degradation in acidic environments. Conversely, in basic conditions (pH > 7), the surface undergoes deprotonation, leading to a negatively charged surface that repels the anionic EY, which decreases adsorption efficiency and impedes the photodegradation process. The repulsive forces diminish the effective contact between the dye and the photocatalyst, ultimately reducing the degradation rate. Additionally, the pH of the solution can influence the dispersion or agglomeration of CeO_2_ nanoparticles; extreme acidic or basic conditions tend to promote aggregation, which limits the available surface area for photocatalytic activity. At pH 5, the CeO_2_ nanoparticles can achieve a stable dispersion, striking an optimal balance between surface charge, minimal aggregation, and high adsorption affinity for the anionic EY dye, thus facilitating efficient photocatalytic degradation under these conditions.

##### 3.4.1.2 Impact of CeO_2_ dose on the degradation of EY

The dosage of photocatalysts is a critical factor that can greatly influence photocatalytic efficiency. Increasing the amount of photocatalyst is anticipated to accelerate the photofading reaction rate due to the higher availability of photocatalyst for generating electron–hole pairs and adsorbing dyes. However, this positive correlation holds only up to a specific dosage; beyond this point, further increases in catalyst dosage can hinder the degradation effectiveness of the photocatalyst. This phenomenon can be attributed to solution opacity, as a higher catalyst concentration leads to turbidity, which reduces photon flux penetration and subsequently diminishes photodegradation efficiency. Additionally, increased turbidity results in light scattering that further impedes the photocatalytic degradation process. Furthermore, at higher dosages, photocatalyst agglomeration may occur, reducing the active surface area and consequently lowering the reaction rate. Agglomeration diminishes the surface area available for photocatalytic reactions due to the formation of large clusters of CeO_2_ nanoparticles, which hinders effective dye adsorption and photon interaction. The reduction in active surface sites of these agglomerated nanoparticles results in a lower degradation efficiency. Additionally, the clustering of CeO_2_ nanoparticles limits photon penetration into deeper layers, thereby reducing the generation rate of electron–hole pairs and resulting in decreased photocatalytic activity. Consequently, this study optimized the dosage of CeO_2_ nanoparticles at a pH of 5 to improve the photocatalytic degradation of EY.

**Fig. 7:**
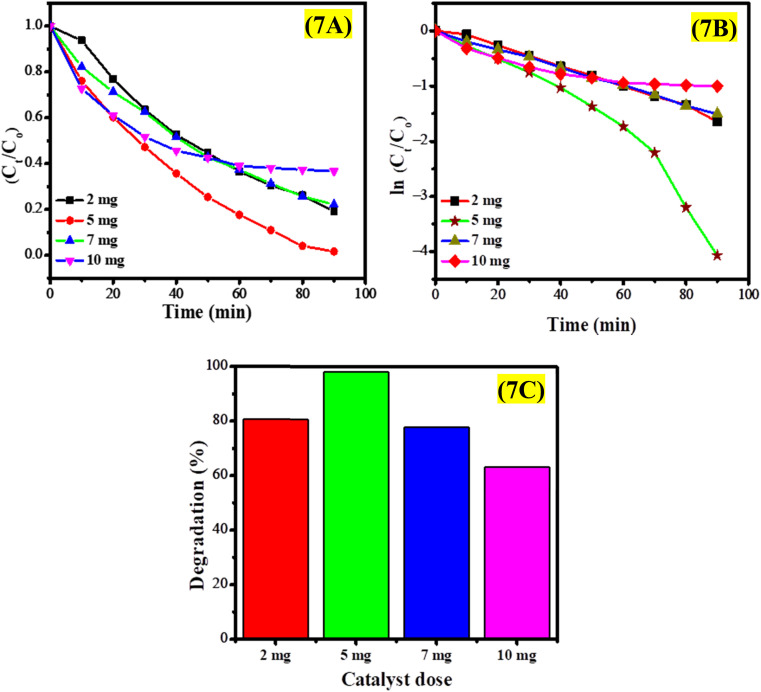
(A) Graph of *C*_*t*_/*C*_o_*versus* time, (B) kinetic study using pseudo-first-order kinetics, and (C) percentage degradation of EY using different amounts of CeO_2_ NPs.

To investigate the effect of CeO_2_ dosage on the photocatalytic degradation of EY, varying amounts (2 mg, 5 mg, 7 mg, and 10 mg) of CeO_2_ nanoparticles were introduced into the EY solution under optimized pH conditions, as depicted in Fig. S11.† The degradation process was observed over a period of 90 minutes for each dosage. The highest degradation rate of 98.3% was achieved with a 5 mg dosage of the photocatalyst, as shown in Fig. S11B.† However, at increased dosages of CeO_2_ nanoparticles, a decline in photocatalytic degradation efficiency was noted (see Fig. S11C and S11D†). The turbidity of the EY solution at higher CeO_2_ concentrations led to light scattering, which adversely affected the photocatalytic degradation process. Fig. 7A illustrates the photocatalytic degradation profile of EY utilizing CeO_2_ nanoparticles at varying catalyst dosages. The data indicates that higher dosages of CeO_2_ enhance the degradation rate of EY, with the optimal degradation occurring at a catalyst dose of 5 mg. The kinetic analysis for the photodegradation of EY with varying catalyst amounts is displayed in Fig. 7B. While Fig. 7C presents the percentage degradation of EY over time in a bar graph format. The photocatalytic performance of CeO_2_ nanoparticles has been evaluated against existing literature concerning the photodegradation of eosin yellow. The data indicates that CeO_2_ nanoparticles exhibit superior efficiency compared to other photocatalysts in the degradation of EY (Table 1).

**Table 1:** Comparison of the photocatalytic efficiency of CeO_2_ NPs to other photocatalysts for the degradation of EY

Catalyst	Time (minutes)	Light source	Degradation (%)	Ref.
ZnO	90	UV lamp (15 W)	92	48
Co-doped ZnO-g-C_3_N_4_	180	Port 9600 full-spectrum solar simulators (equipped with 150 W ozone-free Xenon lamps) and filtered through a 420 nm dichroic UV filter	96	49
Nd-ZnO–MWCNTs	180	Port 9600 full-spectrum solar simulators (equipped with 150 W ozone-free Xenon lamps) and filtered through a 420 nm dichroic UV filter	80	50
TiO_2_@TPPS	50	Visible light (300 W)	99	51
Zn_2_SnO_4_–V_2_O_5_	180	Tungsten lamp (150 W)	92	52
Fe–NiO	180	UV light (254 nm)	93.7	53
Polyaniline–TiO_2_	180	UV light	99.8	54
CeO_2_	90	Solar light	99	This work

#### 3.4.2 Free radical trapping experiment and degradation mechanism

**Fig. 8:**
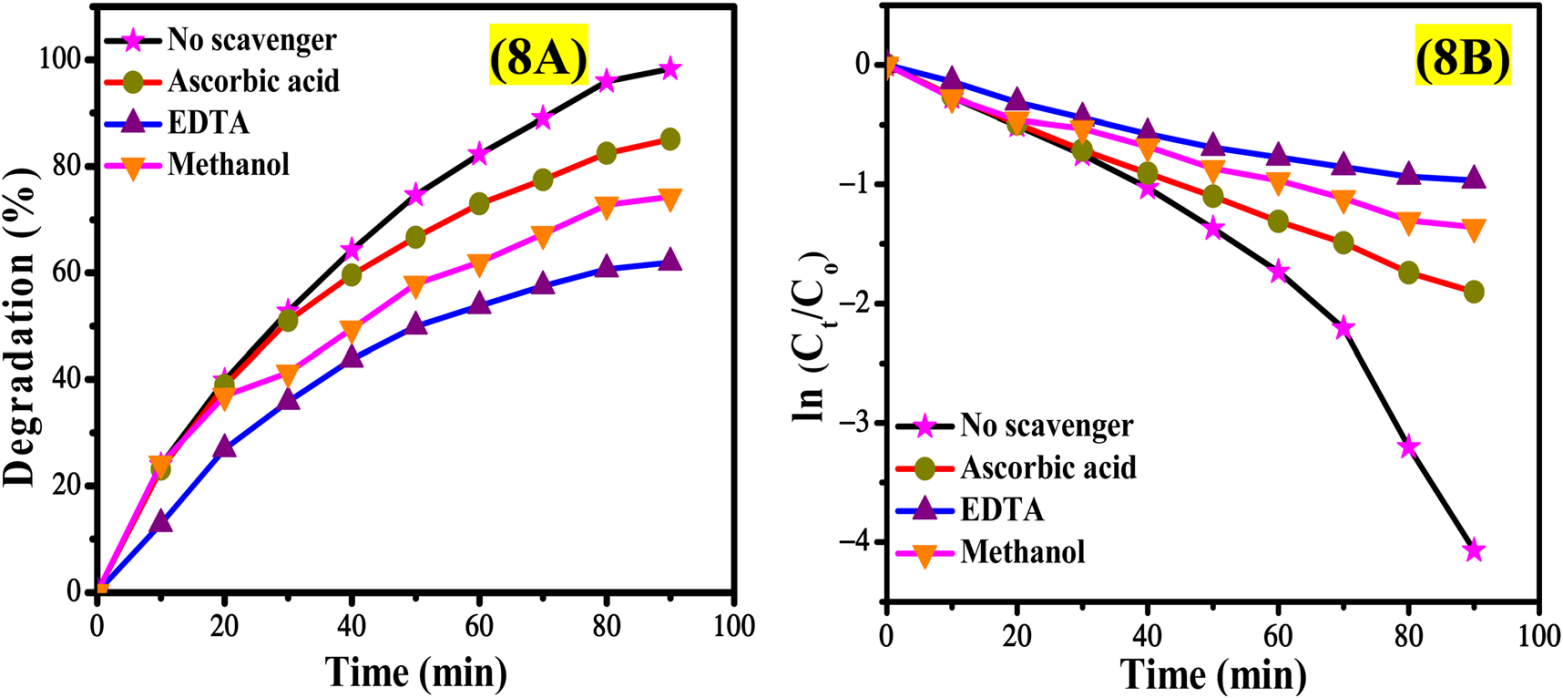
The influence of the radical scavenger on the (A) degradation percentage and (B) the rate constant of the photocatalytic degradation of EY.

Free radicals play a significant role in the photodegradation of dyes, facilitating the initiation and propagation of chemical reactions that result in structural alterations and color fading of the dye. To investigate the influence of free radicals on the photocatalytic degradation of EY, a trapping experiment was performed utilizing ascorbic acid to capture superoxide free radicals (O_2_˙^−^), EDTA to scavenge holes (h^+^), and methanol to neutralize hydroxyl radicals (˙OH). The degradation of EY, illustrated in Fig. S12,† was analyzed through UV spectra in the presence of various radical scavengers. Notably, the effectiveness of CeO_2_ nanoparticles decreased by 13% when ascorbic acid was included in the reaction system, compared to the degradation observed without any radical scavenger (Fig. S12B†). The introduction of EDTA into the photocatalytic system markedly reduced the degradation efficiency of CeO_2_ nanoparticles, resulting in a 38% inhibition, indicating that photogenerated holes are crucial for the degradation of EY, as demonstrated in Fig. S12C.† Similarly, the addition of methanol to the EY solution containing CeO_2_ nanoparticles led to a significant decline in photocatalytic performance, with photodegradation rates dropping from 98% (without trapping agents) to 74%. This observation underscores the important role of photogenerated ˙OH in the photocatalytic removal of EY, as shown in Fig. S12D.† The rate constant for EY photodegradation, detailed in Table S3,† further illustrates the impact of trapping agents, revealing that radical scavengers considerably diminished the photocatalytic efficiency of CeO_2_ nanoparticles, as depicted in Fig. 8A and B. The results of the radical trapping experiment indicated that the primary active species involved in the photocatalytic degradation of EY are photogenerated h^+^ and O_2_˙^−^. The h^+^ produced through photoinduction can directly oxidize water pollutants, resulting in the formation of ˙OH, which subsequently enhances indirect oxidation *via* ˙OH. The proposed mechanism for the photocatalytic degradation of EY is illustrated in the equations provided below.

6 CeO_2_ + *hν* → CeO_2_(h_νb_^+^ + e_cb_^−^)

7 h_νb_^+^ + H_2_O → HO˙ + H^+^

8 h_νb_^+^ + HO^−^ → HO˙

9 O_2_ + e_cb_^−^ → O_2_˙^−^

10 O_2_˙^−^ + HO˙ + EY → Degradation products

when exposed to solar light, electrons in the valence band of CeO_2_ nanoparticles are promoted to the conduction band, resulting in the formation of a hole (h^+^), as described in eqn (6). The generated h^+^ subsequently interacts with hydroxyl ions and water, producing highly reactive hydroxyl radicals (˙OH) as indicated in eqn (8). Concurrently, the excited electrons engage with molecular oxygen, yielding superoxide anions (O_2_˙^−^) as shown in eqn (9). These reactive species subsequently target the EY, facilitating its degradation into carbon dioxide and water.

## 4. Conclusions

The voltammetric detection of eosin yellow dye utilizing COOH-fMWCNTs/GCE, alongside the photocatalytic degradation of EY through CeO_2_ nanoparticles synthesized *via C. fistula*, marks notable progress in both analytical and environmental fields. The COOH-fMWCNTs/GCE exhibited remarkable efficacy in EY dye detection, attributed to its high surface area, superior adsorption properties, and effective electron transfer capabilities. Optimization of the parameters further led to improved sensitivity of COOH-fMWCNTs/GCE, achieving a detection limit of 1.29 nM for EY. Conversely, employing *C. fistula* extract for the biosynthesis of CeO_2_ nanoparticles provided an eco-friendly and effective method for the photodegradation of EY, with XRD analysis confirming the formation of face-centered cubic nanocrystals with an average crystallite size of 5.44 nm. Optical analysis through UV-vis spectroscopy confirmed the formation of nano-sized CeO_2_ nanoparticles with a band gap energy of 3.24 eV. The photoluminescence spectrum exhibited multiple emission bands, with the most prominent peak observed at a wavelength of 609 nm. Scanning electron microscopy revealed the agglomeration of nanoparticles into a cluster-like structure, a typical approach to reduce surface energy. Additionally, energy-dispersive X-ray spectroscopy indicated a cerium content of 69.5%, affirming the synthesized sample's purity. Under optimized conditions, specifically a solution pH of 5 and a catalyst dosage of 5 mg, a maximum dye degradation efficiency of 99% was achieved within 90 minutes using the phytosynthesized CeO_2_ nanoparticles as a photocatalyst. To further elucidate the photocatalytic degradation mechanism, free radical trapping experiments were conducted that proved the involvement of the electron holes to be the major contributor in the degradation process. Overall, it is concluded that the combination of advanced detection and degradation methods offers a comprehensive approach to detect, manage and mitigate the adverse impacts of dyes such as EY.

## Data availability

The authors declare that the data are available in this manuscript in the form of tables and figures.

## Conflicts of interest

There is no conflict of interest to declare.

## Supplementary Material

RA-015-D4RA08231A-s001
