# Supplementary material for: Nanosensor for the detection of eosin yellow and its photocatalytic degradation using phytosynthesized cerium oxide nanoparticles
Source: RSC Adv. 2025 Feb 6;15(6):4095–110. doi: 10.1039/d4ra08231a (PMC11801179; doi:10.1039/d4ra08231a)
Supplement: RA-015-D4RA08231A-s001 [file RA-015-D4RA08231A-s001.pdf]

Supplementary Information (SI) for RSC Advances.
This journal is © The Royal Society of Chemistry 2025

Nanosensor for the Detection of Eosin Yellow and its Photocatalytic Degradation Using Phytosynthesized Cerium Oxide Nanoparticles

Muhammad Usman Sadiq[1,2], Afzal Shah[1*], Anum Zahid[3], Adeela Javaid[2], Faiza Jan Iftikhar[4]

[1]Department of Chemistry, Quaid-i-Azam University, Islamabad, 45320, Pakistan

[2]Department of Chemistry, The University of Azad Jammu and Kashmir, Muzaffarabad, 13100, Pakistan

[3]Department of Chemistry, Pir Mehr Ali Shah Arid Agriculture University, Rawalpindi, 46000, Pakistan

[4]National University of Technology (NUTECH), Islamabad 44000, Pakistan

*Correspondence: afzals_qau@yahoo.com (Afzal Shah)

Table S1. Parameters of EIS obtained from Nyquist plots of bare and COOH-*f*MWCNTs/GCE

	R_s (Ω)	R_{ct} (Ω)	Z_w ($Ss^{1/2}$)	C_{dl} (F)	α
**Bare GCE**	118.2	6.28E+03	135.9E–06	2.19E–06	0.79
**COOH-*f*MWCNTs/GCE**	120.3	1.04E+03	171.9E–06	3.11E–06	0.88

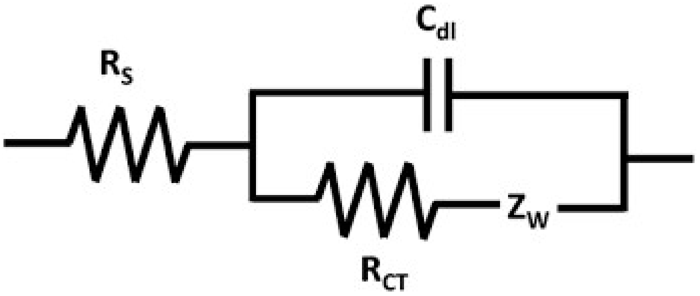

Figure S1. Randle's equivalent circuit used for the calculation of parameters from electrochemical impedance spectra.

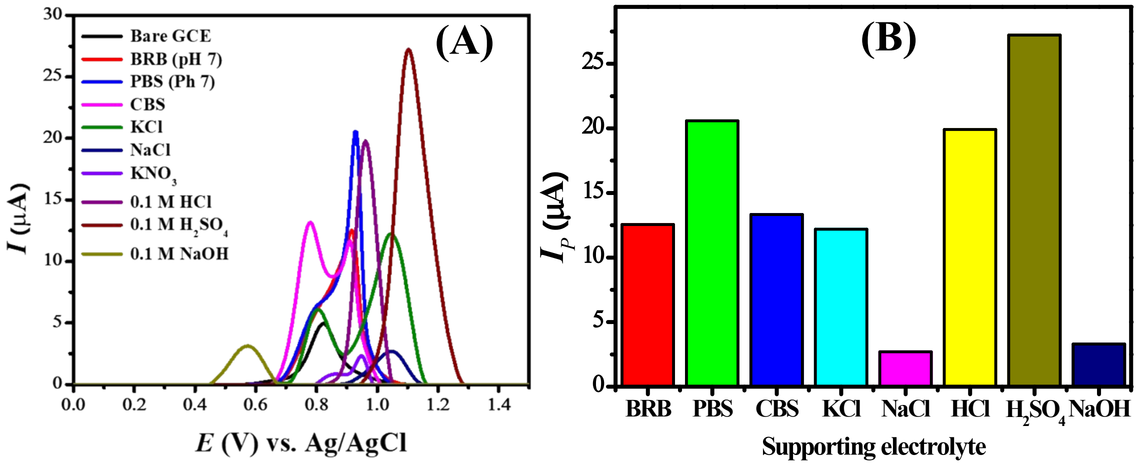

Figure S2. (A) SWV of 100 µM EY in different supporting electrolytes (B) bar graph showing peak current versus supporting electrolyte.

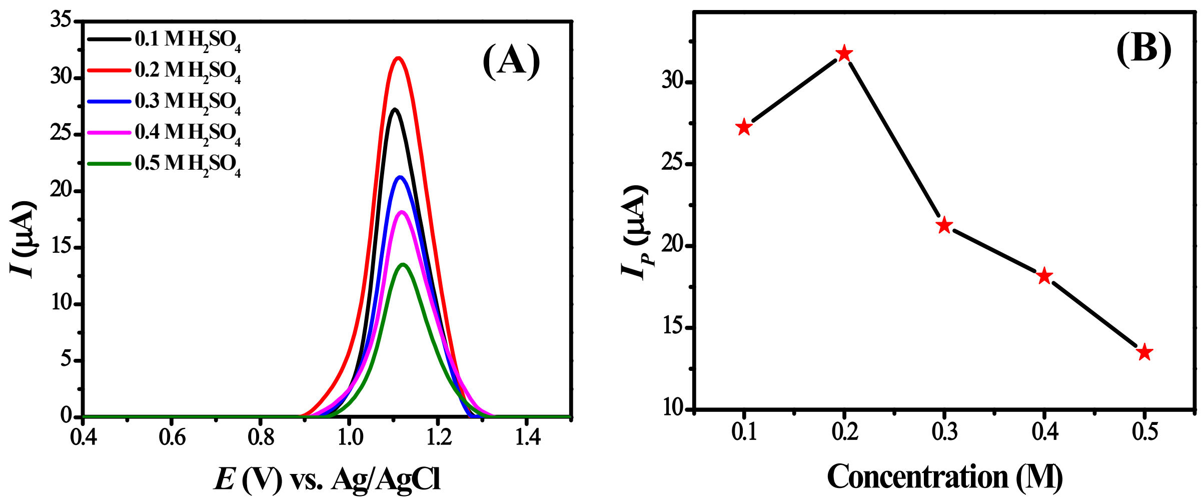

Figure S3. (A) Influence of concentration of H_2SO_4 on peak current of 100 μM EY and (B) plot showing peak current versus concentration of H_2SO_4.

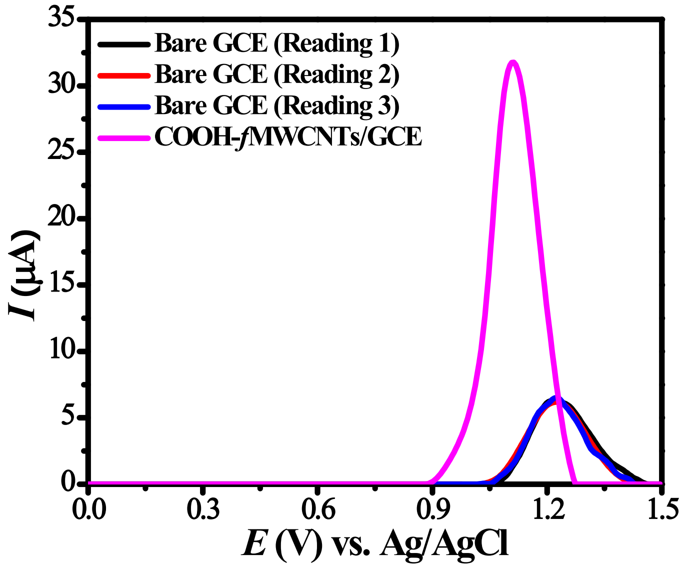

Figure S4. SWV response of EY at bare GCEs and COOH-*f*MWCNTs using 0.2 M H_2SO_4 as supporting electrolyte.

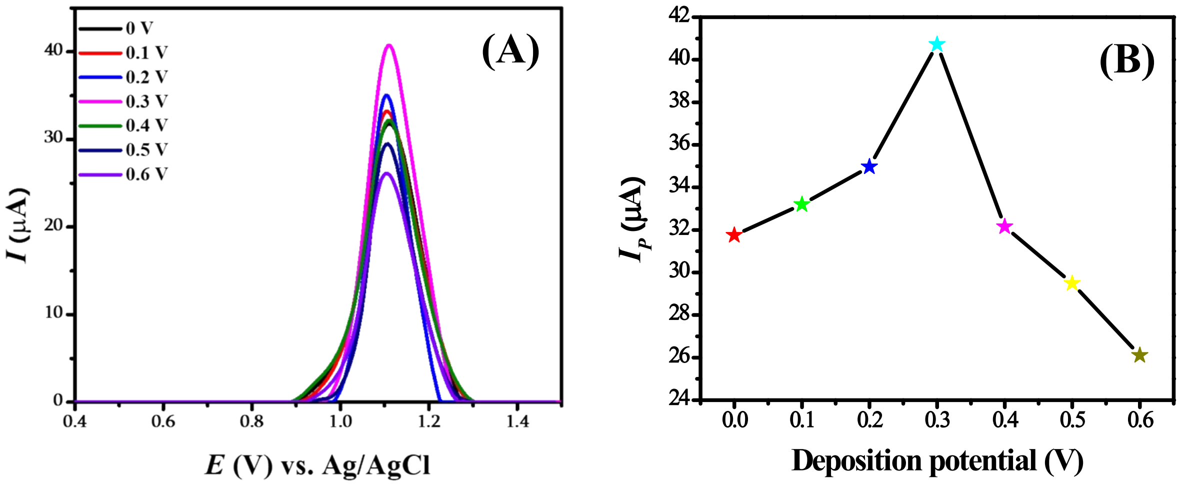

Figure S5. (A) SWV of 100 μM EY under various applied deposition potential and (B) plot of peak current versus deposition potential.

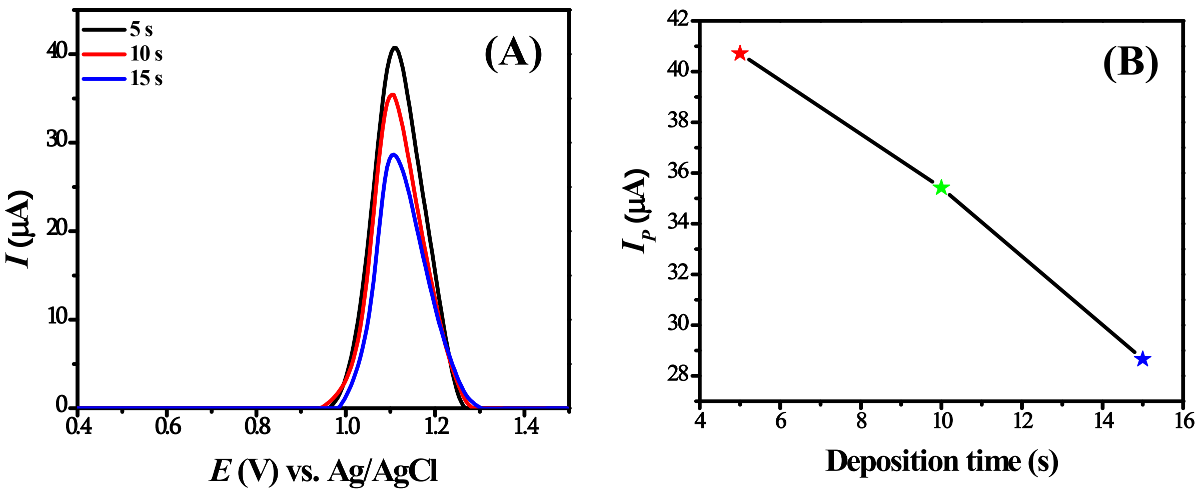

Figure S6. (A) Impact of deposition time on the peak intensity of EY using COOH/*f*MWCNTs/GCE and (B) Plot of I_p (μA) versus deposition time.

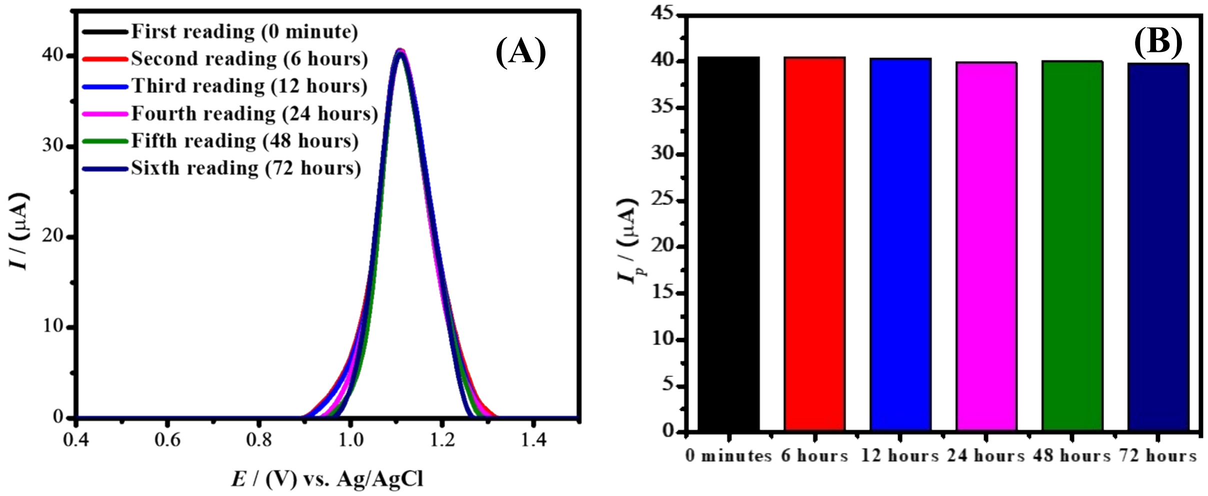

Figure S7. (A) SWV of 100 μM EY recorded under optimized conditions after various time period (B) bar graph showing peak current response of 100 μM EY.

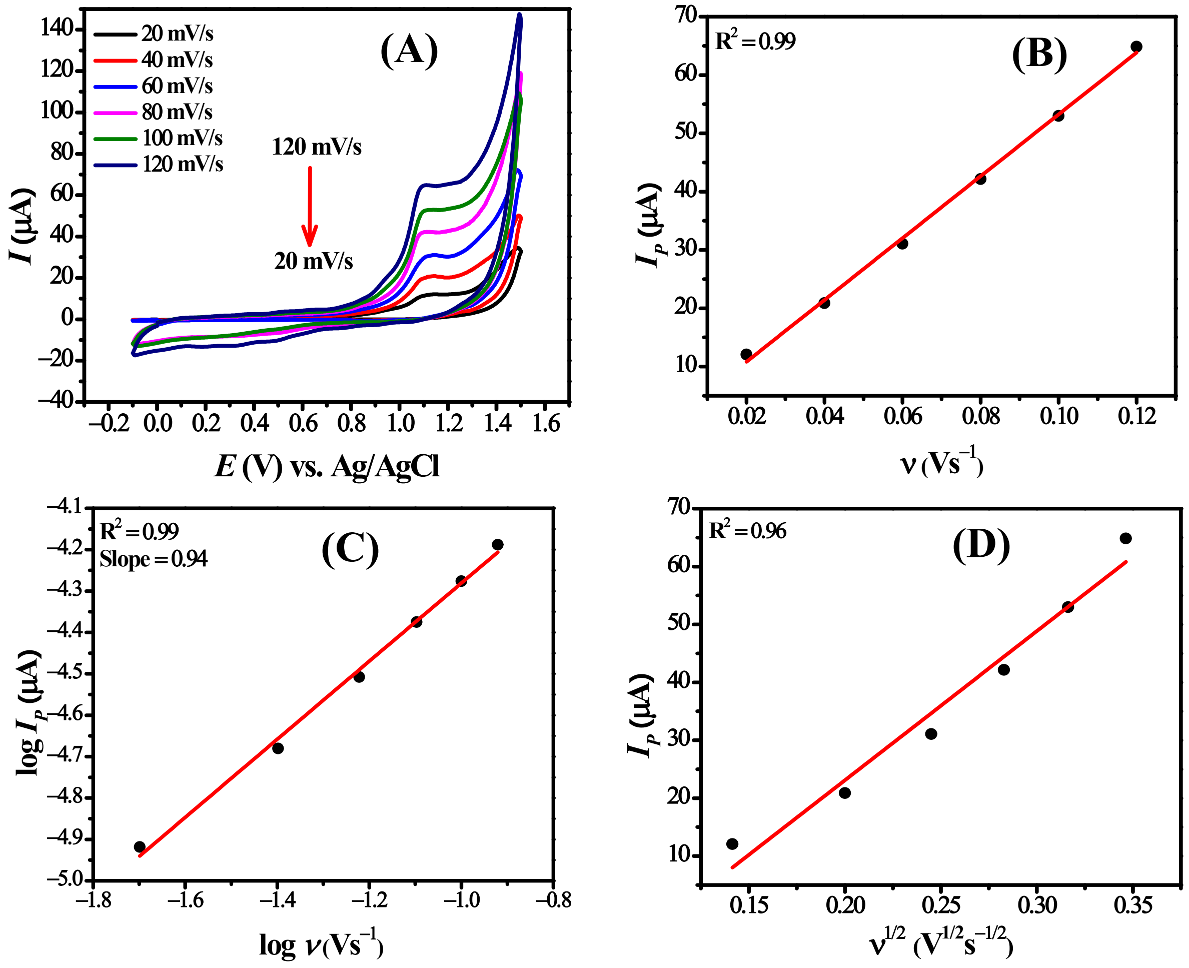

Figure S8. (A) Effect of various scan rates on the anodic peak current of EY in supporting electrolyte of 0.2 M H_2SO_4; (B) Plot between I_p vs. υ; (C) log I_p vs. log υ, and (D) Peak current vs. square root of scan rate.

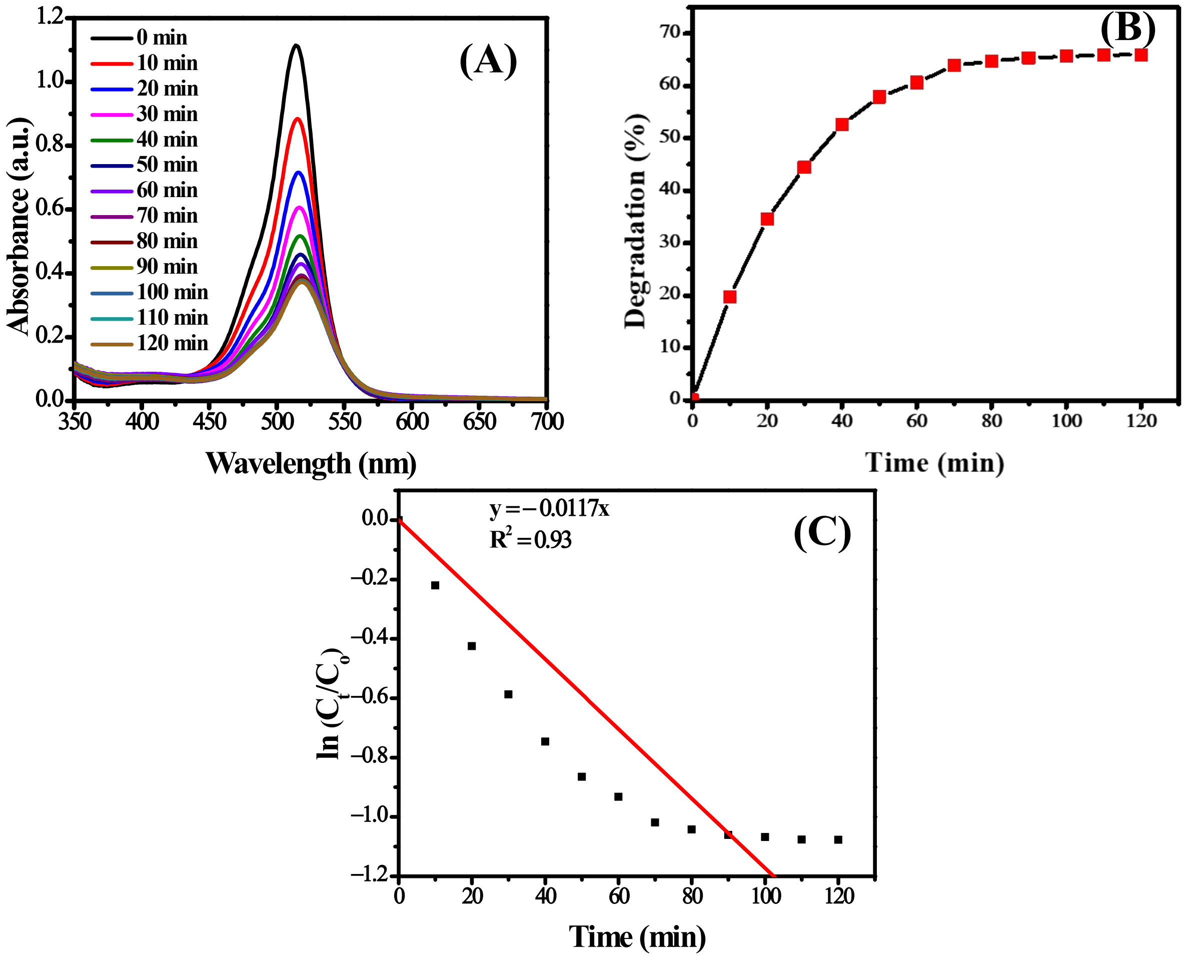

Figure S9. (A) Absorption spectra showing degradation of EY with time using 2 mg dose of CeO_2 NPs under pH 7 media, (B) plot showing extent of absorption with time, and (C) pseudo-first order kinetic plot of EY degradation.

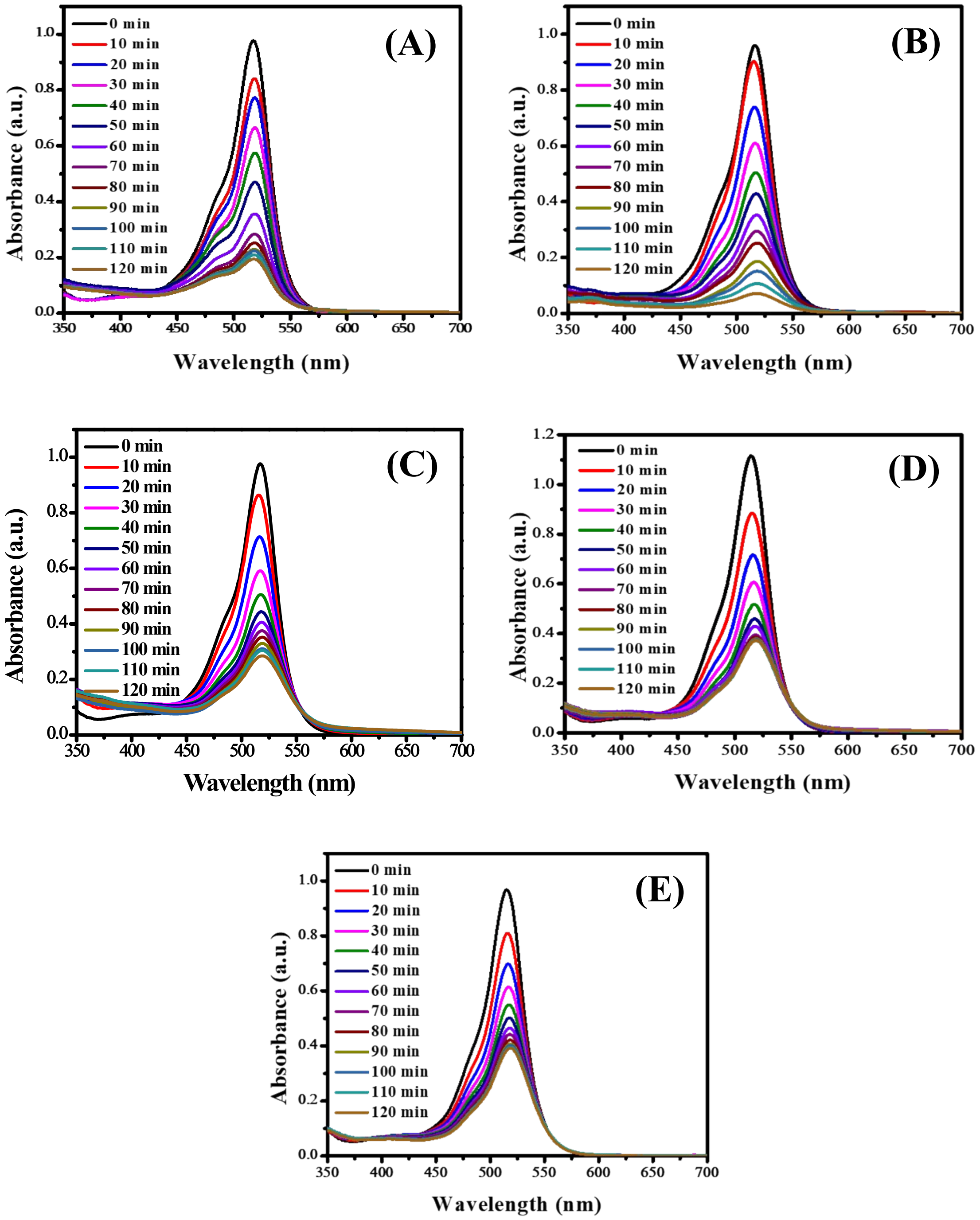

Figure S10. UV-visible spectra showing photocatalytic degradation of EY in solutions of various pH levels: (A) pH 4, (B) pH 5, (C) pH 6, (D) pH 7, and (E) pH 8.

Table S2. Rate constants of the photocatalytic degradation of EY at different pH.

pH	Rate constant (min^{-1})
4	0.0149
5	0.0188
6	0.0125
7	0.0117
8	0.0094

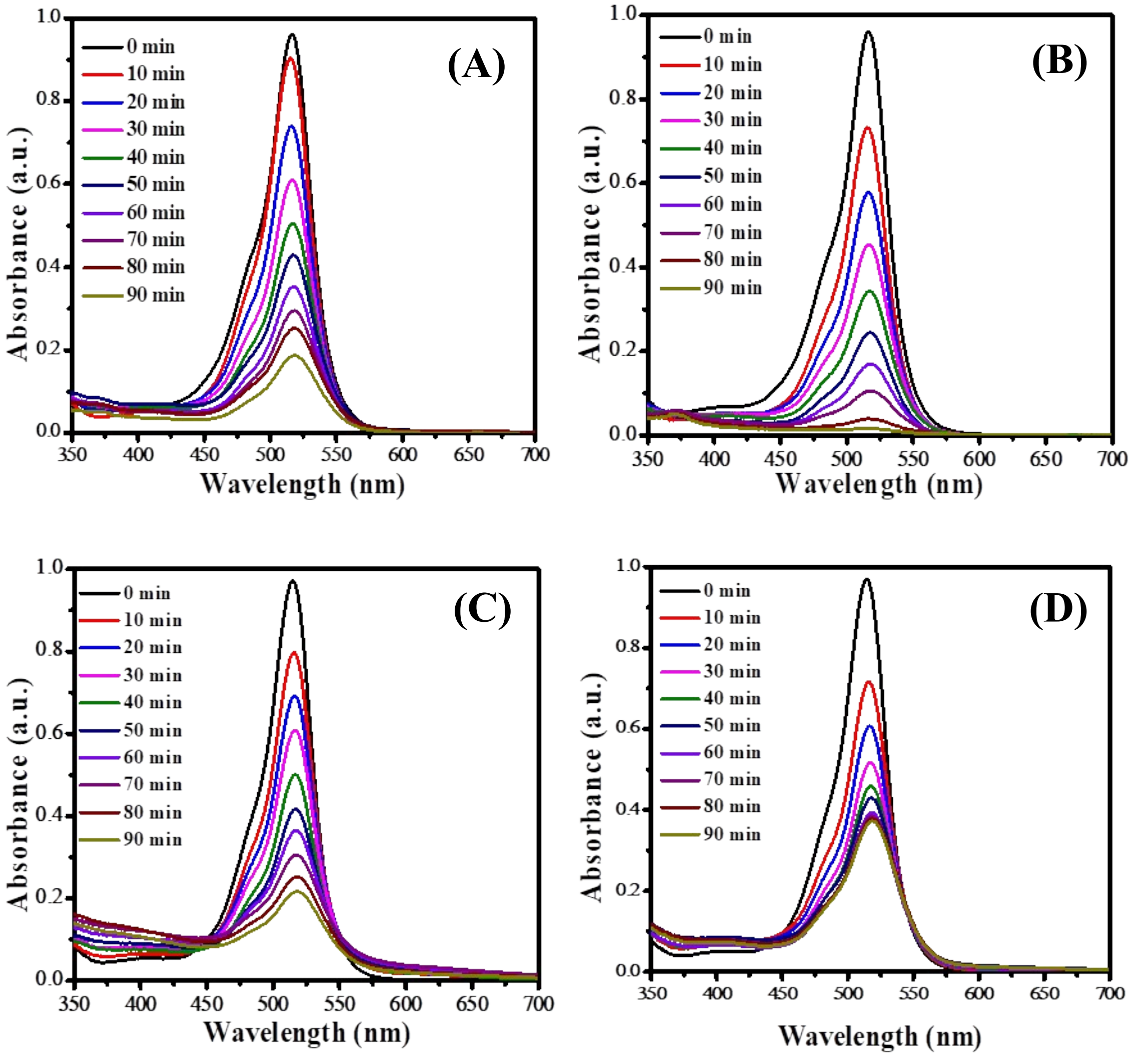

Figure S11. Photocatalytic degradation of EY conducted using varying doses of CeO_2 nanoparticles, specifically (A) 2 mg, (B) 5 mg, (C) 7 mg, and (D) 10 mg, in an environment with a pH of 5.

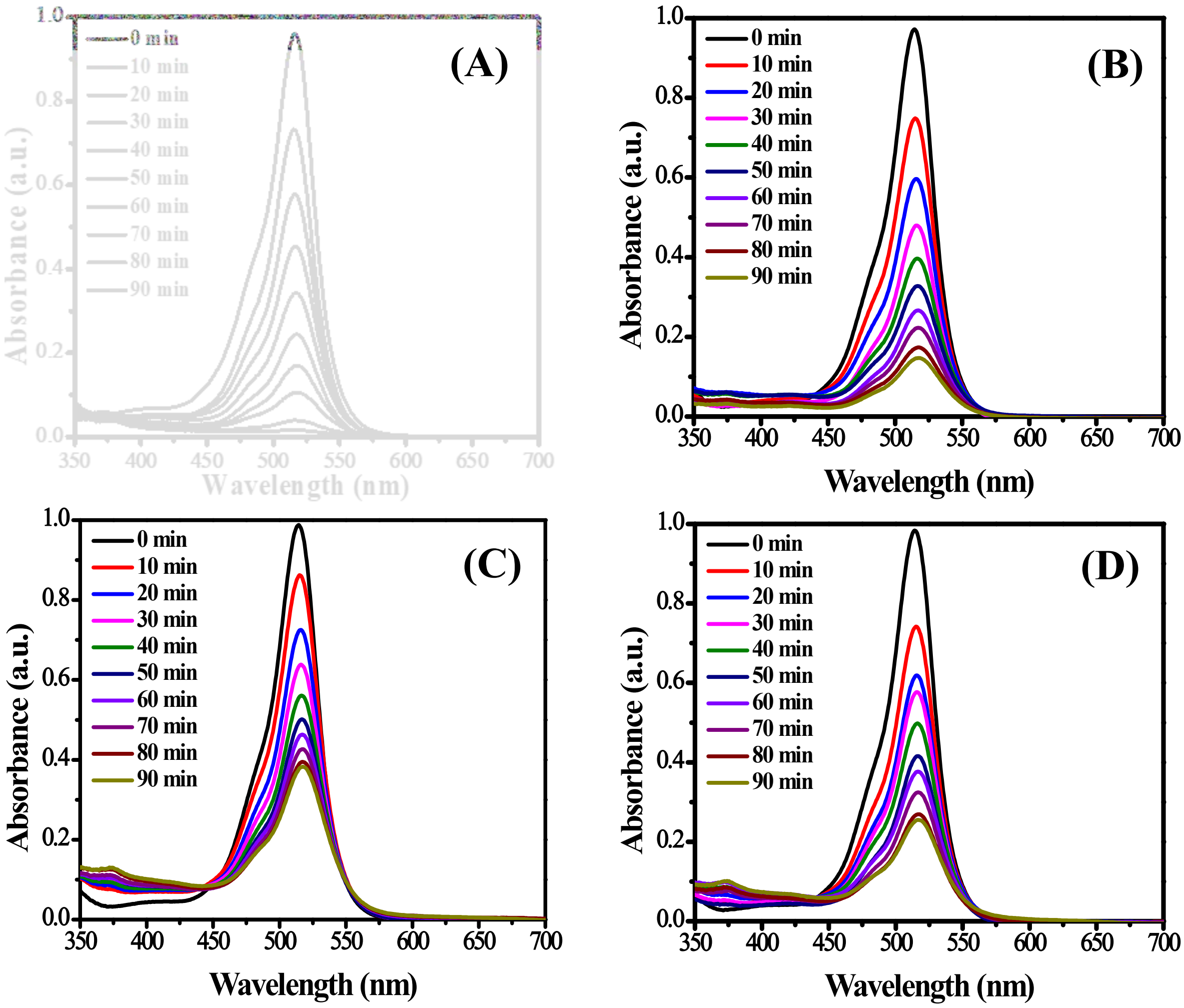

Figure S12. Photodegradation of EY using CeO_2 NPs (A) without free radical scavenger, (B) with ascorbic acid, (C) with EDTA, and (D) with methanol.

Table S3. Rate constants of photocatalytic degradation of EY with and without free radical scavengers.

**Scavenger**	**Rate constant (min^{-1})**
No scavenger	0.0360
Ascorbic acid	0.022
EDTA	0.0121
Methanol	0.0161
